# Extracellular vesicle-associated repetitive element DNAs as candidate osteosarcoma biomarkers

**DOI:** 10.1038/s41598-020-77398-z

**Published:** 2021-01-08

**Authors:** Linda Cambier, Kevin Stachelek, Martin Triska, Rima Jubran, Manyu Huang, Wuyin Li, Jianying Zhang, Jitian Li, David Cobrinik

**Affiliations:** 1 The Vision Center and The Saban Research Institute, Children’s Hospital Los Angeles, 4650 Sunset Blvd, MS163, Los Angeles, CA 90027 USA; 2 Cancer Biology and Genomics Program, Keck School of Medicine, University of Southern California, Los Angeles, CA 90089 USA; 3 Division of Hematology/Oncology, Children’s Hospital Los Angeles, Los Angeles, CA 90027 USA; 4 Henan Luoyang Orthopedic Hospital (Henan Provincial Orthopedic Hospital)/Henan Provincial Orthopedic Institute, 82 Qiming Road, Luoyang, 471002 Henan China; 5 Department of Biological Science, The University of Texas at El Paso, El Paso, TX 79968 USA; 6 Department of Ophthalmology and Roski Eye Institute, Keck School of Medicine, University of Southern California, Los Angeles, CA 90089 USA; 7 Department of Biochemistry and Molecular Medicine and Norris Comprehensive Cancer Center, Keck School of Medicine, University of Southern California, Los Angeles, CA 90089 USA

**Keywords:** Cancer, Cell biology

## Abstract

Osteosarcoma (OS) is the most common malignant bone tumor in children and young adults. Despite that high-risk factors have been identified, no test for early detection is available. This study aimed to identify circulating nucleic acid sequences associated with serum extracellular vesicle (EV) preparations at the time of OS diagnosis, as a step towards an OS early detection assay. Sequencing of small nucleic acids extracted from serum EV preparations revealed increased representation of diverse repetitive element sequences in OS patient versus control sera. Analysis of a validation cohort using qPCR of PEG-precipitated EV preparations revealed the over-representation of *HSATI*, *HSATII*, *LINE1-P1*, and *Charlie 3* at the DNA but not RNA level, with receiver operating characteristic (ROC) area under the curve (AUC) ≥ 0.90. *HSATI* and *HSATII* DNAs co-purified with EVs prepared by precipitation and size exclusion chromatography but not by exosome immunocapture, indicative of packaging in a non-exosomal complex. The consistent over-representation of EV-associated repetitive element DNA sequences suggests their potential utility as biomarkers for OS and perhaps other cancers.

## Introduction

Osteosarcoma (OS) is the most common malignant bone tumor of children, adolescents, and young adults, representing approximately 1% of newly diagnosed cancers in adults, and 3–5% in children^1,2^. With current treatment regimens, patients with non-metastatic OS have 5-year survival rates above 65% whereas the ~ 25% of patients presenting with metastases have a 5-year survival of less than 20%^3,4^. As such, early detection of OS prior to metastasis could significantly improve outcomes.

Early detection is especially needed in individuals who are predisposed to OS either genetically or through iatrogenic exposures. OS occurs at increased rates in several monogenic hereditary cancer syndromes such as retinoblastoma (*RB1*)^5^, Li-Fraumeni syndrome (*TP53*), Bloom syndrome (*RECQL2*), Werner syndrome (*RECQL3*), and Rothmund-Thomson syndrome (*RECQL4*). OS also occurs with increased frequency in children exposed to radiation or alkylating agents, in Diamond–Blackfan anaemia patients and in adults with bone disorders such as Paget’s Disease. Combined genetic predisposition and exposure to DNA damaging agents confers particularly high risk; for example, relative risk for children with hereditary retinoblastoma increased from ~ 69 without such treatments to ~ 302 for radiotherapy and ~ 539 for radiotherapy plus chemotherapy in the largest treatment-stratified analysis^5^.

The need for OS biomarkers is reflected in a large yet inconclusive literature. Early studies focusing on bone markers such as alkaline phosphatase showed highly variable increases in OS patients^6^. Later proteomic studies revealed two as-yet uncharacterized OS-associated proteins^7^ whereas studies of miRNAs showed variable results^8–10^. A recent study identified 56 miRs that were upregulated in pre-treatment OS patient plasma^11^; however, among the top candidates (*miR-21*, *miR-221*, and *miR-106a*), levels increased by only ~ 2.4–8-fold and sensitivity was at best ~ 85%. An alternative approach is to detect aneuploidy via cell-free DNA (cfDNA) whole genome sequencing, yet at present this has limited sensitivity^12^ due to the dilution of tumor with non-tumor cfDNA. Currently no biomarkers have been shown to reliably detect naïve pre-symptomatic OS in predisposed individuals^13^.

In this study, we aimed to identify circulating biomarkers that distinguish OS patients from healthy controls, as a step towards a liquid biopsy for early OS detection. Liquid biopsies may detect circulating tumor components including cfDNA, tumor cells, and extracellular vesicles (EVs)^14–19^, a category that includes exosomes, shedding vesicles, microparticles, retroviral-like particles, ectosomes, microvesicles, oncosomes, and apoptotic bodies^20,21^. EVs are released by most if not all cells^22^ and carry components of their cell of origin such as proteins, lipids, metabolites, and various types of RNA^23,24^. Among the different types of EVs, exosomes and oncosomes are more highly produced by cancer cells than by normal cells, are often present at increased levels at cancer diagnosis, may further increase during tumor progression^15^, and carry cargo that reflects metastatic progression and treatment response^25,26^. Moreover, EV preparations may contain exosomal as well as non-exosomal tumor components. Thus, we aimed to identify OS biomarkers in serum-derived EV preparations.

To identify EV-associated OS biomarkers we compared the abundance of nucleic acid sequences in OS patient versus control serum EV preparations. Specifically, we sequenced small nucleic acids extracted from EV preparations and examined differential representation of unique as well as repetitive element sequences which are often produced and may be released by cancer cells^27^, including by OS cells^28^. We then evaluated whether the same sequences were differentially represented in different patient cohorts and by different EV isolation and analytic methods. Through these approaches we identified circulating EV-associated repetitive element DNA sequences that were more abundant in OS sera compared to healthy sera in two patient cohorts.

## Results

### Over-representation of repetitive element sequences in OS EV preparations

**Figure 1:**
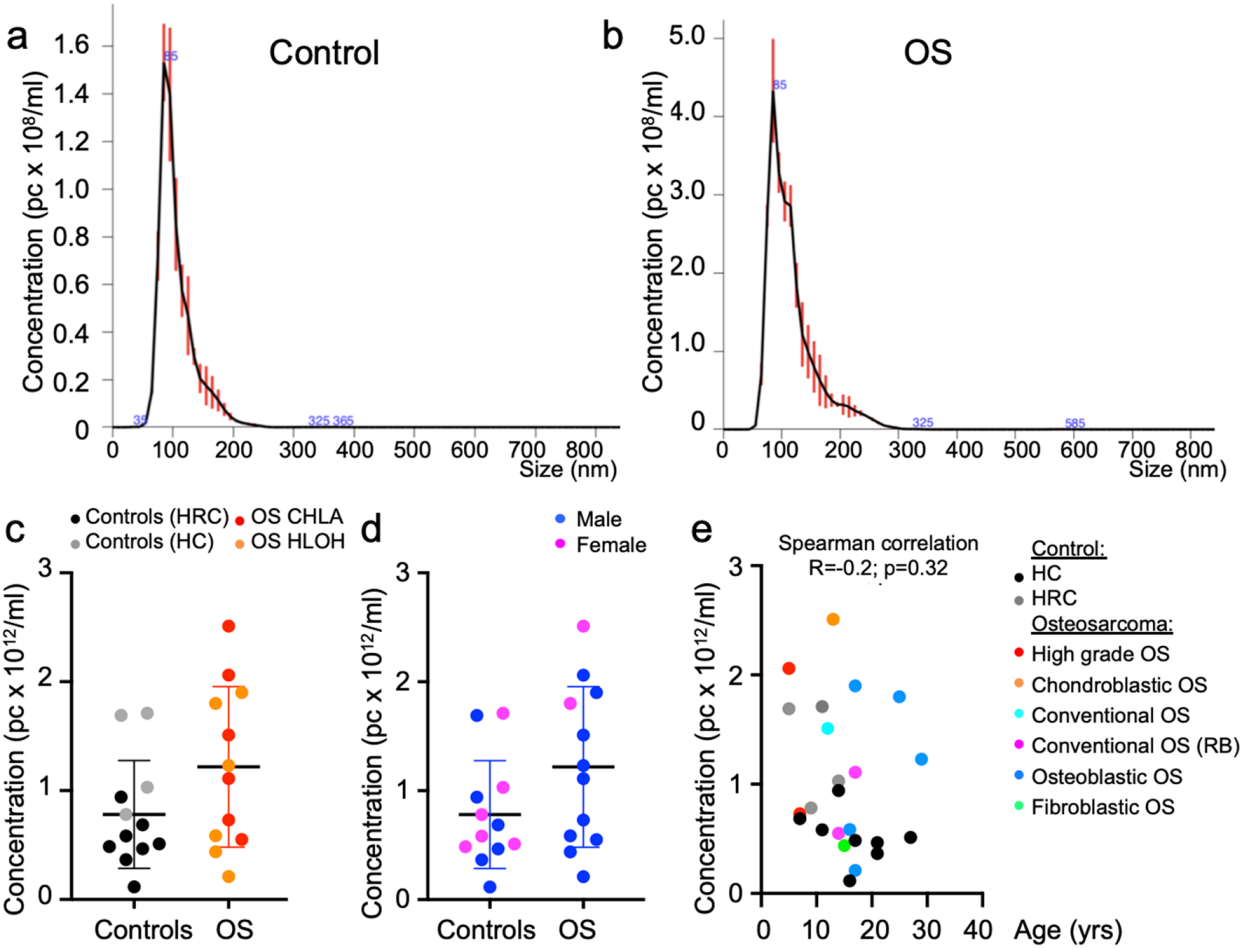
Patient EV preparation characteristics. (**a**,**b**) Representative control (**a**) and OS (**b**) particle size distributions in preparations used for nucleic acid extraction and sequencing, as defined by nanoparticle tracking (NanoSight). (**c**,**d**) Dot plots representing EV concentration of preparations from sera of OS patients (n = 12) and controls from hereditary retinoblastoma sibling (HRC, n = 4), and unrelated healthy (HC, n = 8) and color-coded by source (**c**) and sex (**d**). Lines represent mean and standard deviation. Groups were compared using two**‐**tailed, unpaired, Mann Whitney U test; *p < 0.05. (**e**) Dot plots of EV concentration versus donor age and color coded according to OS type. Spearman’s correlation (r) between EV concentration and donor age was not significant (*p* = 0.32).

To identify OS biomarkers, we compared nucleic acid sequences associated with EV preparations from sera of OS patients and healthy controls. Initial analyses were performed on a discovery cohort of treatment-naïve OS patients from Children’s Hospital Los Angeles (CHLA) and Henan Luoyang Orthopedic Hospital (HLOH), comprised of males and females between 5 and 29 years old and presenting with different OS types. Control cohorts were comprised of healthy siblings of hereditary retinoblastoma patients who had not developed retinoblastoma (hereditary retinoblastoma controls; HRCs) and unrelated approximately age-matched healthy individuals (healthy controls; HC) (Table 1a). EV preparations were made with the commercial ExoQuick kit based on polyethylene glycol (PEG) precipitation, with recognition that EV as well as non-EV components are isolated^29,30^. Nanoparticle tracking analysis of each sample revealed similar size distributions of control and OS EVs between 50 and 150 nm, which is characteristic of exosomes (Fig. 1a,b). EV concentrations were not significantly higher in sera from OS patients compared to controls (Fig. 1c). Likewise, EV concentrations were similar in OS patient serum from USA (CHLA) and China (HLOH) and for different patient ages, genders, and OS types (Fig. 1c–e).

**Table 1:** Osteosarcoma patient and control donor characteristics.

Source	Sample	Gender	Age (yrs)	OS type
**a. Discovery cohort (nucleic acid sequencing)**				
*Osteosarcomas*				
CHLA	OS-C1	M	7	High grade, extensive necrosis
	OS-C2	F	13	Chondroblastic, necrosis
	OS-C3	M	12	Conventional
	OS-C4	M	5	High grade, small cell variant
	OS-C7	M	17	Conventional secondary to RB
	OS-C8	M	14	Conventional secondary to RB
HLOH	OS-H6	M	16	Osteoblastic
	OS-H7	M	17	Osteoblastic
	OS-H11	F	25	Osteoblastic
	OS-H19	M	15	Fibroblastic
	OS-H20	M	17	Osteoblastic
	OS-H27	M	29	Osteoblastic
* Controls*				
CHLA	HC1	F	17	
	HRC1	M	5	
	HRC2	F	14	
	HRC3	F	11	
	HRC4	F	9	
HLOH	HH12	M	10	
	HH17	M	14	
	HH21	M	21	
	HH23	M	16	
	HH25	M	27	
	HH28	F	21	
	HH29	F	11	
**b. Validation cohort (qPCR)**				
*Osteosarcomas*				
CHLA	OS-C1	M	7	High grade, extensive necrosis
	OS-C3	F	12	Conventional
	OS-C5	M	13	Conventional
	OS-C9	M	19	Conventional
HLOH	OS-H21	M	43	High grade, extensive necrosis
	OS-H24	M	46	Osteoblastic
	OS-H25	F	16	Osteoblastic
	OS-H34	M	14	Osteoblastic
*Controls*				
CHLA	HC2	F	38	
	HC3	M	38	
IR	HI1	M	20	
	HI2	M	19	
	HI3	F	20	
	HI4	F	18	
	HI5	M	20	
	HI6	M	19	
	HI7	M	20	
	HI8	F	18	
	HI9	F	19	
	HI10	F	20	

OS-C, osteosarcoma from CHLA; OS-H, osteosarcoma from HLOH; HC, healthy control from CHLA; HH, Healthy control from HLOH; HI, healthy control from Innovative Research Inc.; HRC, hereditary retinoblastoma sibling control.

To detect differentially represented EV-associated RNA and DNA sequences, nucleic acids were extracted from OS and control EV preparations using SeraMir small RNA enrichment kit (SBI) without DNase treatment and a sequencing library was built by addition of a 5′-RNA adapter and a 3′-DNA adapter followed by PCR amplification and sequencing. Comparison of uniquely mapped sequences using DESeq2^31^ identified 107 significantly over-represented genes and 587 significantly under-represented genes (≥ twofold change, *p*.adj < 0.05) in OS samples (Supplementary Fig. 1). However, the over-represented sequences had poor OS sensitivity and specificity (not shown). As a far greater proportion of genes were under-represented in OS samples, we considered whether our analysis of uniquely mapped sequences ignored potentially relevant over-represented non-uniquely mapped sequences. Thus, we evaluated the differential representation of the major repetitive element categories after aligning reads to RepeatMasker^32^. This indicated that OS serum EV preparations had greater representation of almost all repetitive element categories including the most abundant LINE1, LTR/ERV, α-satellite and SINE/Alu categories but not the low complexity ribosomal RNA and scRNA sequences (Supplementary Fig. 2A, B). The most highly represented sequence was the LINE1 family member *L1P1* (Supplementary Fig. 2C).

**Figure 2:**
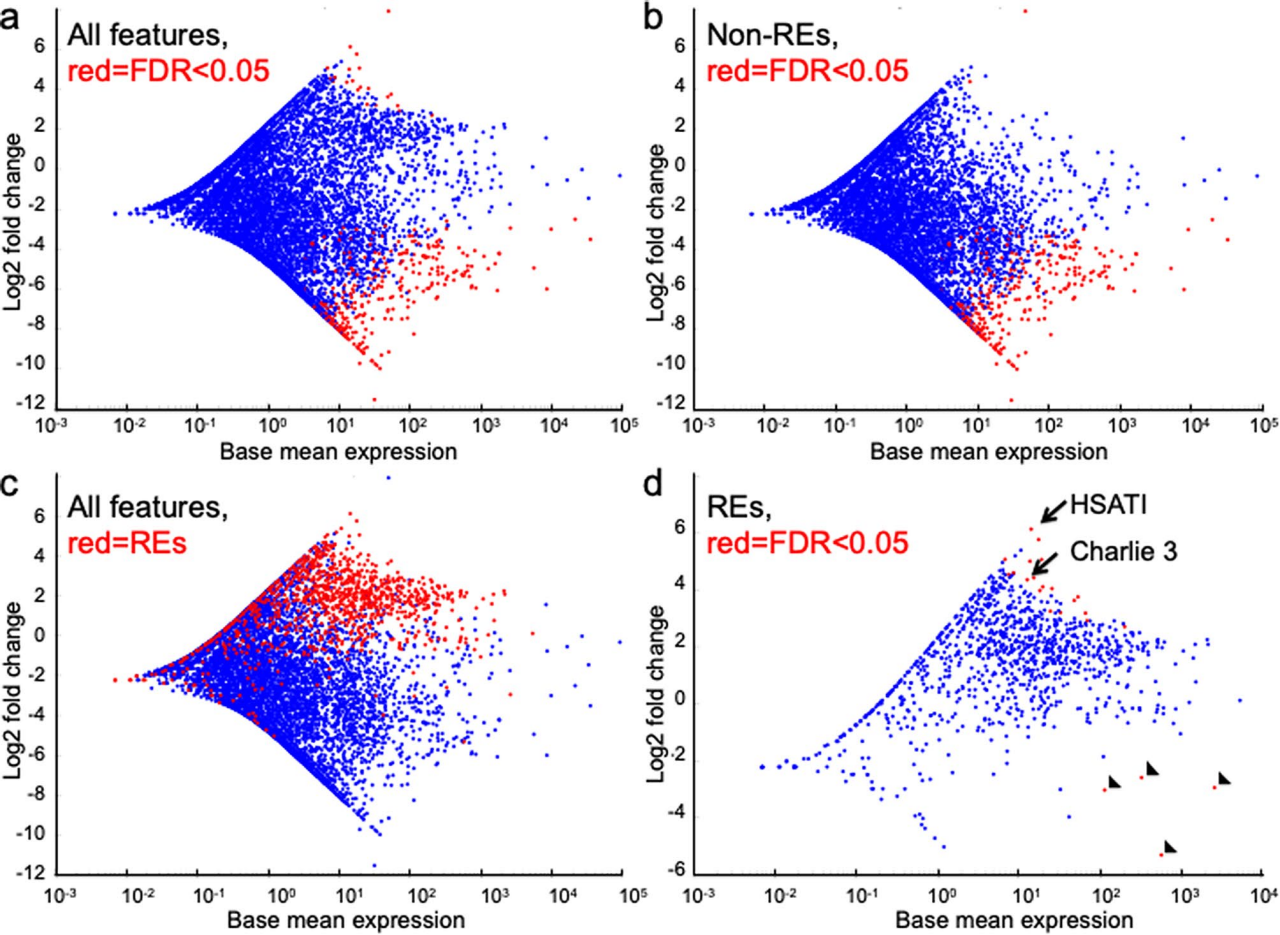
Over-representation of repetitive elements in OS EV–associated sequences. MA plot for sequence features differentially represented in control and OS serum EV preparations as defined by TEtranscripts analysis 1. (**a**) Differentially represented single-copy genes and repetitive elements (REs), significantly differentially represented in red. (**b**) Differentially represented single-copy genes, significantly differentially represented in red. (**c**) Differentially represented single-copy genes in blue and REs in red. (**d**) Differentially represented REs, significantly differentially represented in red. Arrows, the significantly over-represented *HSATI* and *Charlie 3*. Arrowheads, significantly under-represented REs. Significantly differentially represented defined by FDR < 0.05, Wald test.

While these analyses revealed consistent over-representation of repetitive element sequences in OS serum EV preparations, the identities of the most over-represented elements were uncertain since programs that are not specifically designed for repetitive element detection may erroneously map repetitive element reads^33^. To more accurately define the differential repetitive element representation, sequences were evaluated with TEtranscripts, which maps repetitive element sequences more accurately and quantitatively than non-dedicated programs^34^. Using default settings with reads mapped to the GRCh38 genome, TEtranscripts confirmed that OS samples had far more significantly under-represented than over-represented sequences in comparison to control samples (Fig. 2a) and showed that most of the under-represented sequences were single-copy genes (Fig. 2b). In contrast, a vast proportion of repetitive element sequences were over-represented in OS EV preparations (Fig. 2c) albeit with only 19 significantly over-represented versus four significantly under-represented (Fig. 2d). Among significantly over-represented repeat elements, Human Satellite I (*HSATI*) had the highest fold change (log_2_ (6.14), *p*.adj = 0.007) (Fig. 2d and Table 2, Analysis 1).

**Table 2:** Repeat elements significantly differentially represented in OS versus control EV preparations (*p*.adj < 0.05; elements examined in the validation cohort in bold italics).

Analysis 1 (mapped to GRCh38)	log_2_FC	*p*.adj	Analysis 2 (mapped to hg19)	log_2_FC	*p*.adj
***HSATI:Satellite:Satellite***	***6.14***	***0.007***	***HSATII:Satellite:Satellite***	***2.73***	***0.002***
LTR85a:Gypsy:LTR	5.77	0.003	L1MA6:L1:LINE	2.65	0.041
LTR75:ERVL:LTR	5.07	0.042	MSTC:ERVL-MaLR:LTR	2.52	0.001
LTR16E2:ERVL:LTR	5.07	0.001	(GAATG)n:Satellite:Satellite	2.51	0.035
LTR16A1:ERVL:LTR	5.01	0.014	Charlie4z:hAT-Charlie:DNA	2.37	0.011
Tigger8:TcMar-Tigger:DNA	4.60	0.025	Harlequin-int:ERV1:LTR	2.28	0.032
MLT1H2-int:ERVL-MaLR:LTR	4.54	0.016	L1M3:L1:LINE	2.05	0.038
BLACKJACK:hAT-Blackjack:DNA	4.43	0.029	L1MB8:L1:LINE	2.03	0.005
***Charlie3:hAT-Charlie:DNA***	***4.36***	***0.030***	L1PB4:L1:LINE	2.00	0.021
MER113:hAT-Charlie:DNA	4.11	0.019	L1MC3:L1:LINE	1.90	0.046
MLT1A1-int:ERVL-MaLR:LTR	4.05	0.015	L1PB2:L1:LINE	1.89	0.026
L1MCb:L1:LINE	3.98	0.037	L1MEc:L1:LINE	1.88	0.047
MLT2B2:ERVL:LTR	3.75	0.018	MLT1A0:ERVL-MaLR:LTR	1.81	0.016
HSAT4:centr:Satellite	3.63	0.025	L1MB7:L1:LINE	1.80	0.028
LTR32:ERVL:LTR	3.25	0.042	L1MDa:L1:LINE	1.45	0.041
Charlie4z:hAT-Charlie:DNA	3.20	0.034	L1PA12:L1:LINE	− 2.27	0.002
CER:Satellite:Satellite	3.18	0.021			
MLT1E2:ERVL-MaLR:LTR	2.92	0.029			
L1MC4a:L1:LINE	2.71	0.040			
MER74A:ERVL:LTR	− 2.61	0.041			
L1PA16:L1:LINE	− 2.96	0.029			
FAM:Alu:SINE	− 3.05	0.015			
FordPrefect:hAT-Tip100:DNA	− 5.34	< 0.001			

Because GRCh38 contains numerous alternative assemblies that are enriched for repetitive elements that might siphon repetitive element reads, adds synthetic centromeric repeat sequences, and hard-masks certain centromeric and genomic repeat arrays^35^, we considered whether these features might affect the ability to detect differential representation of unique or repetitive element sequences. To address this possibility, we re-performed TEtranscripts analysis with reads aligned to hg19, which lacks the GRCh38 alternative assemblies. This identified 15 significantly over-represented repeat elements, of which Human Satellite II (*HSATII*) had highest fold change (log_2_ (2.73), *p*.adj = 0.002), and one significantly under-represented element in OS versus control sequences (Table 2, Analysis 2 and Supplementary Fig. 3). The significantly differentially represented repetitive elements identified using hg19 had little overlap with those identified when mapping to GRCh38.

### Validation of over-representation of repeat elements in OS EV preparations

We next examined whether the increased representation of repetitive elements was evident in a validation set of mostly distinct samples. The validation cohort consisted of treatment-naïve OS patients from CHLA and HLOH including males and females between 7 and 46 years old and presenting with various OS types as well as approximately age-matched healthy individuals (Table 1b). The validation cohort was independent of the discovery cohort except for re-analysis of OS1 and OS3, which were the only samples with a sufficient quantity to re-test. To assess the repetitive element over-representation, EV-associated nucleic acids were isolated and evaluated using methods that differed from the discovery cohort analyses: EVs were isolated by PEG6000 precipitation^36^ instead of ExoQuick, nucleic acids were extracted using the miRNeasy micro RNA extraction kit (Qiagen) instead of SeraMir, and repetitive elements were examined by reverse transcription and quantitative PCR (RT-qPCR) instead of sequencing. Similar to the discovery cohort, EV concentrations were not significantly higher in sera from OS patients compared to controls (data not shown).

RT-qPCR was used to analyze four representative repetitive element categories including the *HSATI* and *HSATII* satellite sequences that were most differentially over-represented in TEtranscripts Analyses 1 and 2 (Table 2), the *LINE1 P1* family member (*L1P1)* that showed the highest fold change in the RepeatMasker analysis (Supplementary Fig. 2), and *Charlie 3*, another over-represented repetitive element with a significant log_2_ fold change of 4.36 (20.5-fold increase) in TEtranscripts Analysis 1 (Table 2, Fig. 2d). RT-qPCR reactions yielded the predicted product sizes for *HSATI* (406 bp), *L1P1* (83 bp), and *Charlie 3* (104 bp). RT-qPCR of *HSATII* yielded prominent products of 85, 134, 183 and 281 bp, in agreement with *HSATII* genomic structure (Supplementary Fig. 4), instead of a reported ~ 200 bp amplicon found by RT-PCR with the same primers in an OS cell line^37^. The *HSATII* and *Charlie 3* products were confirmed to represent the predicted sequences by Sanger sequencing.

**Figure 3:**
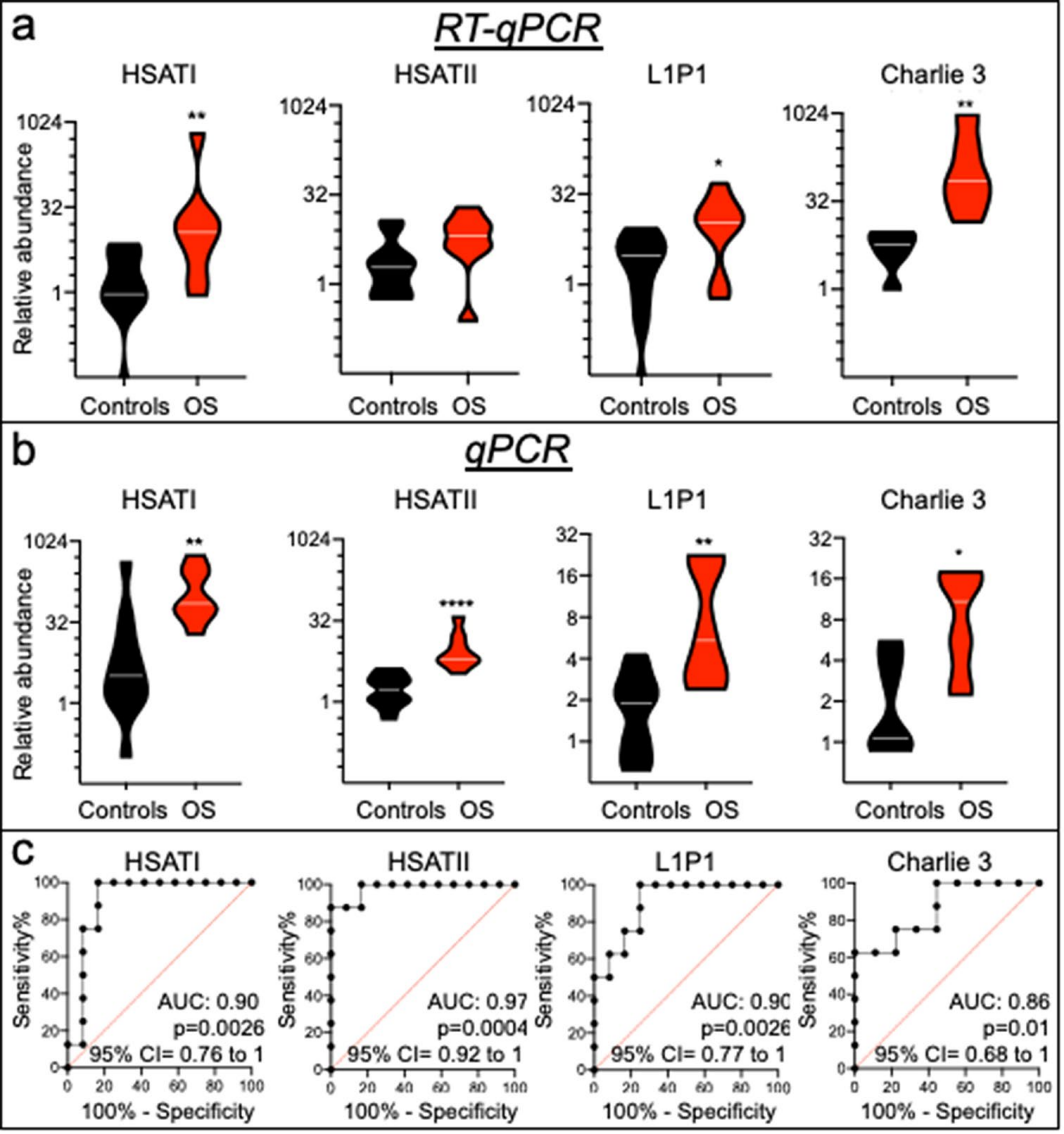
Over-representation of repetitive elements in OS compared to control EV preparations in a validation cohort. (**a**) Violin plots representing relative abundance of *HSATI*, *HSATII*, *L1P1* and *Charlie 3* by RT-qPCR of control (n = 6–11) and OS (n = 7–8) serum EV preparations. RT-qPCR was normalized against *C. elegans *external spike-in miR-39–3p RNA. White lines represent median. (**b**) Violin plots representing relative abundance of *HSATI*, *HSATII*, *L1P1* and *Charlie 3* DNA by qPCR, in the absence of reverse transcription, in control (n = 12) and OS (n = 8) serum EV preparations. qPCR was performed on equal proportions of nucleic acid extracted from 200 ul of OS and control serum. White lines represent median. (**c**) Diagnostic value of *HSATI*, *HSATII*, *L1P1* and *Charlie 3* DNA sequences in serum OS preparations. ROC curves were generated using data in (**b**). Groups were compared using two**‐**tailed, unpaired, Mann Whitney U test; *p < 0.05; **p < 0.01; ***p < 0.001.

For each sample, RT-qPCR was performed on the same proportion of total EV nucleic acid extracted from the same serum volume and was normalized against a spike-in RNA. The analyses confirmed the over-representation of *HSATI*, *HSATII*, *L1P1*, and *Charlie 3* sequences in OS EV preparations (12.42-fold, *p* = 0.0040; 3.33-fold, *p* = 0.062; 3.56-fold, *p* = 0.016; 12.6-fold, *p* = 0.0007; respectively) (Fig. 3a). In contrast, the single-copy gene *HECDT2*, chosen on the basis of a 4.67 log_2_ fold change in TEtranscripts analysis, did not show a significant difference (Supplementary Fig. 5A and B), and was deemed to have been spuriously identified, possibly due to biases in the library construction method^38^. The over-representation of *HSATI*, *HSATII*, *L1P1*, and *Charlie 3* sequences was similar after removal of the OS1 and OS3 samples that were also used in the discovery cohort (Supplementary Fig. 6A). Thus, the over-representation of repetitive element sequences initially detected by nucleic acid sequencing was confirmed in a validation cohort using RT-qPCR.

### Over-representation of repetitive element DNA but not RNA in OS EV preparations

As our nucleic acid isolation and analysis methods could detect RNA as well as DNA sequences, we examined the nucleic acid origin of the over-represented repetitive element sequences by performing qPCR without reverse transcription. With this approach, the *HSATI*, *HSATII*, *L1P1* and *Charlie 3* amplification signals were significantly over-represented in OS versus control EVs (22.18-fold, *p* = 0.0015; 3.7-fold, *p* < 0.0001; 2.86-fold, *p* = 0.0015; 10.29-fold, *p* = 0.011; respectively) (Fig. 3b), indicative of differential representation of repetitive element DNA, rather than RNA. Evaluation of the sensitivity and specificity by Receiver Operating Characteristic (ROC) curves yielded area under the curves (AUCs) of 0.86 or greater for each repetitive element (Fig. 3c). As for the RT-qPCR analyses, results were similar after removal of OS1 and OS3 (Supplementary Fig. 6B and C). *HSATI*, *HSATII*, *L1P1* and *Charlie 3* DNAs were similarly increased in OS samples from USA (CHLA) and China (HLOH) and in high-grade as well as non-high grade OS samples (Supplementary Fig. 7). In contrast, qPCR of *IL17RA* and *ZNF3* sequences, which are within 1 Mb of *HSATI* on chromosome 22 (chr22q11.21) and within 40 Mb of *HSATII* on chromosome 7 (chr7q22.1), respectively, did not show a significant difference between OS and control EVs (Supplementary Fig. 8A and B). Thus, major repetitive elements DNAs (*HSATI*, *HSATII*, *L1P1*, *Charlie 3*) were over-represented, whereas single copy genes were not over-represented, in OS EV preparations.

**Figure 4:**
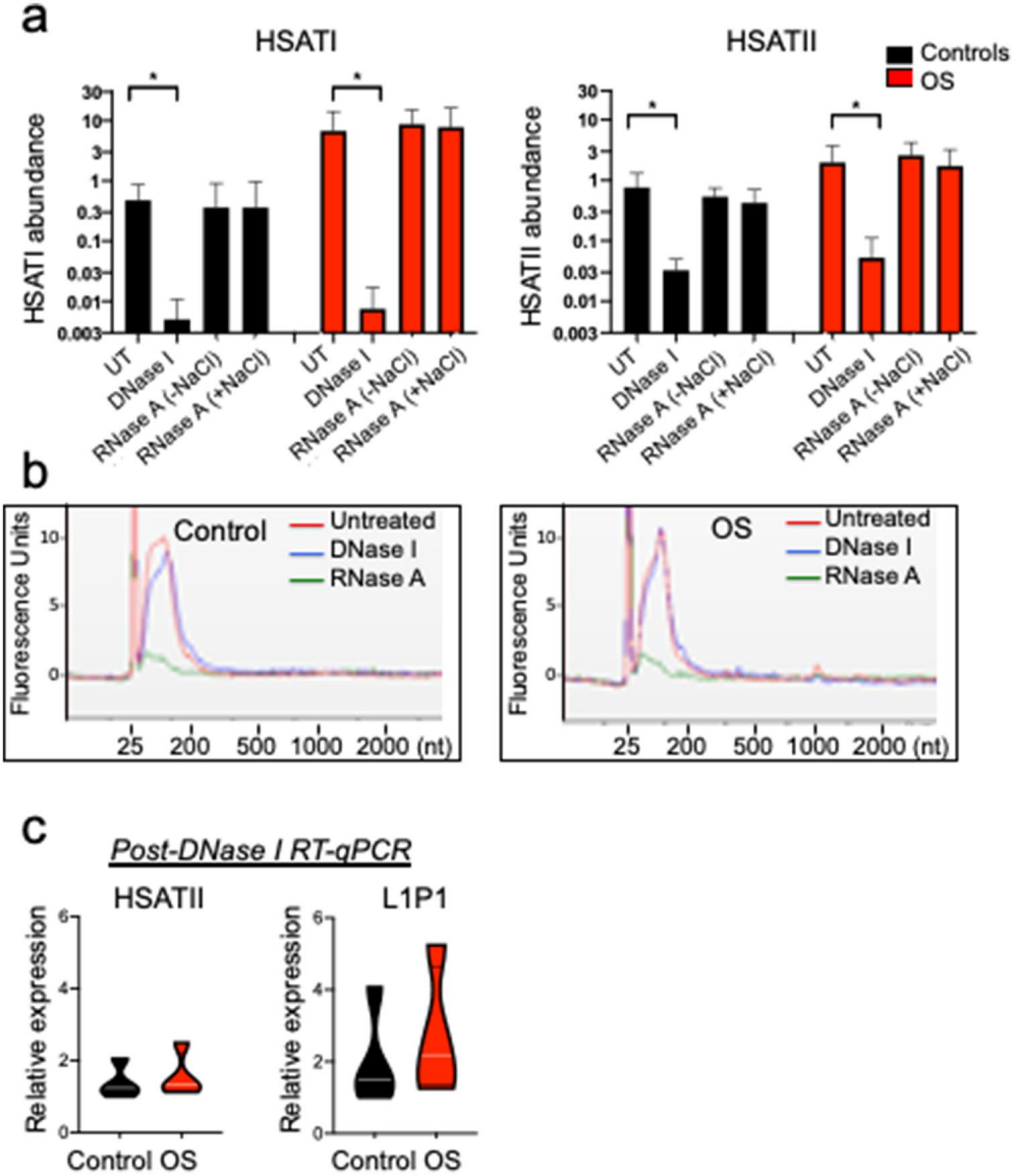
Repetitive element sensitivity to DNase in EV preparations. (**a**) Abundance of *HSATI* and *HSATII* sequences determined by qPCR in control (n = 4) and OS (n = 4) serum EV preparations isolated with PEG and either untreated (UT) or pretreated with DNase I or RNase A with or without NaCl prior to nucleic acid extraction. Treated groups were compared to untreated groups using unpaired Kruskal–Wallis test with uncorrected Dunn’s test where each comparison stands alone; *, *p* < 0.05. Error bars represent standard deviation of biological replicates. (**b**) Bioanalyzer electropherograms of equal proportions of nucleic acids prepared from 200 ul of representative control and OS EV preparations that were untreated or treated with DNase I or RNase A prior to nucleic acid extraction. (**c**) Violin plots representing relative abundance of *HSATII*, *L1P1* and *Charlie 3* by RT-qPCR in control (n = 4) and OS (n = 4) serum EV preparations pre-treated with DNAse I. qPCR was normalized against *C. elegans *external spike-in miR-39–3p RNA. White lines represent median.

To further evaluate the abundance of repetitive element DNAs and control for possible artefactual generation of RT-independent products, PEG-precipitated EV preparations from four OS and four control sera were treated with DNase I or RNase A prior to nucleic acid extraction. RNase A treatments were performed in 1 M NaCl in order to cleave single-stranded RNA as well as in the absence of NaCl in order to cleave single-stranded and double-stranded RNA and RNA strands in RNA-DNA hybrids^39^. After these treatments, nucleic acids were extracted with the miRNeasy Micro kit and *HSATI* and *HSATII* abundance were assessed by qPCR. In these analyses, DNase I treatment eliminated 97–99% of *HSATI* and 80–99% of *HSATII* signals in both OS and control samples, whereas RNase A treatments had no significant effect (Fig. 4a). Bioanalyzer assessments revealed that DNase treatment slightly decreased the amount of nucleic acid whereas RNase A eliminated most but not all of the nucleic acids (Fig. 4b), with the majority of the remaining nucleic acid likely representing protected EV RNA. The repetitive elements’ sensitivity to DNase I prior to nucleic acid extraction implied that the repetitive element DNA sequences were not sequestered inside of EVs.

To assess whether OS serum EV preparations might also have an increased abundance of repetitive element RNAs, PEG-precipitated EVs were prepared, treated with DNase I, and the remaining nucleic acids extracted and examined by RT-qPCR. In these samples, no amplification signal was detected for *HSATI* or *Charlie 3*, while *HSATII* and *L1P1* products were reduced ~ 32–64-fold compared to non-DNase I treated samples and showed no significant difference in control and OS samples (Fig. 4c and data not shown). Thus, *HSATI*, *HSATII*, *L1P1*, and *Charlie 3* DNAs were over-abundant in OS compared to control EV preparations whereas their RNAs were either undetectable or present in similar quantities.

### Co-purification of OS-associated repetitive element DNAs with EVs in size exclusion chromatography but not exosome immunoaffinity capture

To further evaluate if repetitive element DNAs that were more abundant in OS patient PEG-precipitations (here termed ‘OS-associated’ repetitive element DNAs) are associated with EVs, we examined whether they co-purified with EVs prepared by size exclusion chromatography (SEC) and exosome immunoaffinity capture. SEC yields more pure EV populations^40,41^ with lower protein contamination compared to PEG precipitation^42,43^, whereas exosome immunoaffinity capture uses well-characterized surface markers CD9 or CD81 to highly purify intact exosomes^41,44^.

**Figure 5:**
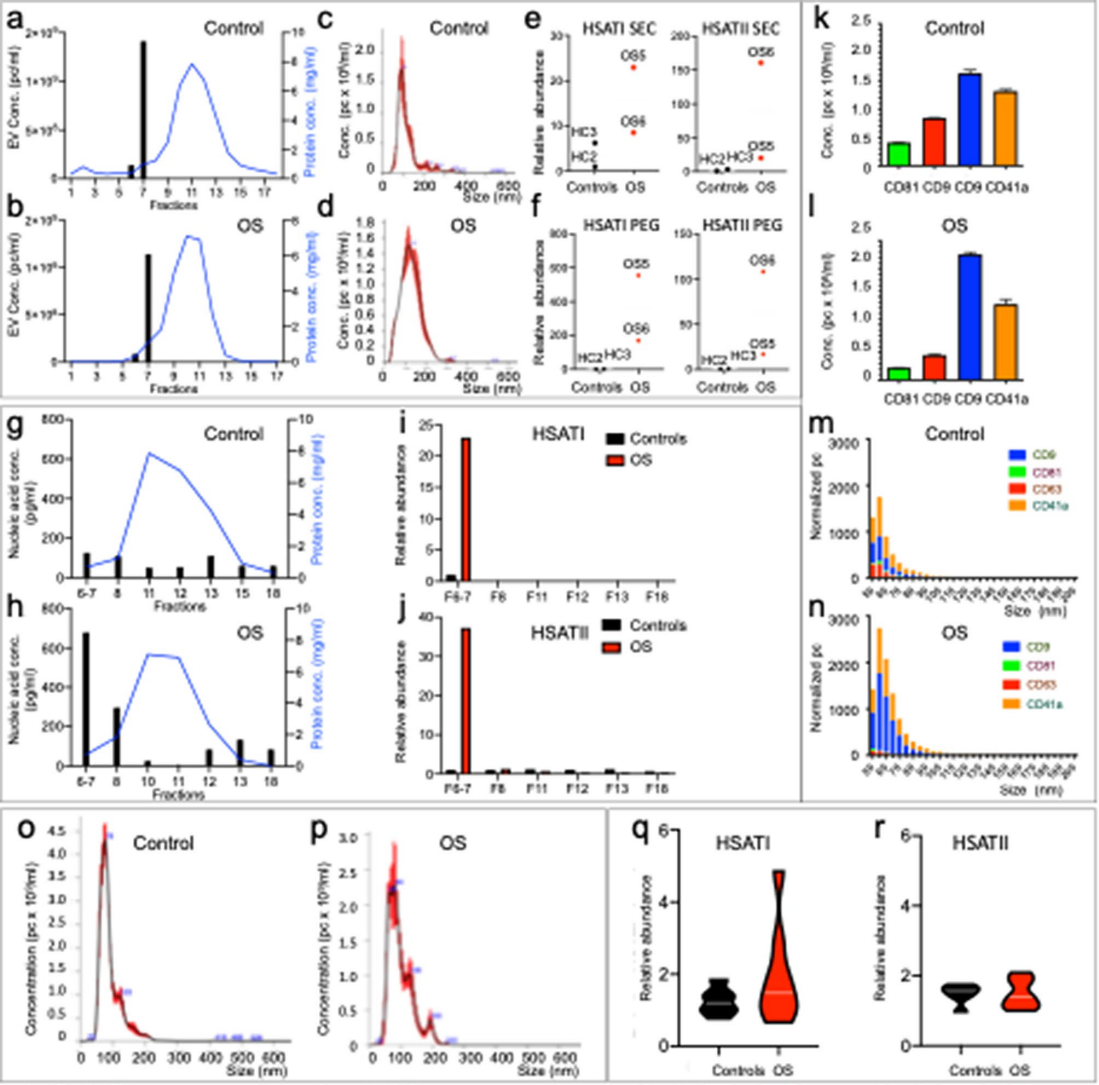
Co-purification of OS-associated repetitive element DNAs with EVs in size exclusion chromatography but not exosome immunoaffinity capture. (**a**,**b**) Representative protein concentration (blue line) and EV concentration (black bars) elution profiles from control (**a**) and OS (**b**) serum separated by size exclusion chromatography (SEC). (**c**,**d**) Representative size distribution of control (**c**) and OS (**d**) serum EV particles in pooled fractions 6 and 7 analyzed by nanoparticle-tracking. (**e**,**f**) Relative abundance of *HSATI* and *HSATII* DNA in two control and two OS SEC (**e**) and PEG (**f**) EV fractions as defined by qPCR. (**g**,**h**) Representative protein concentration (blue line) and nucleic acid concentration (black bars) of pooled EV fraction (F6-7) and selected non-EV fractions (F8, F11, F12, F13 and F18) from the same representative control (**g**) and OS (**h**) SEC separations as in (**a**) and (**b**). (**i**,**j**) Abundance of *HSATI* (**i**) and *HSATII* (**j**) in pooled EV fractions 6–7 and non-EV fractions on one control and one OS SEC analysis as evaluated by qPCR. (**k**–**n**) SP-IRIS analyses by ExoView of EVs isolated by CD9-immunoaffinity capture. (**k**,**l**) Representative concentration of control (**k**) and OS (**l**) fluorescent EV particles immunocaptured on the CD9, CD81 and CD63 antibody spots. Results depict the mean of the measurement of triplicate spots ± SEM, subtracted for IgG spot values and adjusted by dilution factor. (**m**,**n**) Representative size distribution of control (**m**) and OS (**n**) label-free EV particles immunocaptured on the CD9, CD81 and CD63 antibody spots. Results depict the mean of the measurement of triplicate spots ± SEM, subtracted for IgG spot values. (**o**,**p**) Size distribution and particle number of control (**o**) and OS (**p**) EVs isolated by CD9 immunoaffinity capture and analyzed by nanoparticle-tracking. (**q**,**r**) Violin plots representing abundance of *HSATI* (**m**) and *HSATII* (**n**) of control (n = 6) and OS (n = 8) immunoaffinity capture of CD9-positive exosomes evaluated by qPCR. White lines represent median. Groups were compared using two**‐**tailed, unpaired, Mann Whitney U test; *p < 0.05. Similar CD81 immunoaffinity capture results in Supplementary Fig. 11.

We first examined if repetitive element DNAs co-purify with SEC-isolated EVs from two control and two OS samples. Nanoparticle tracking analyses revealed that the control and OS EVs both eluted from size exclusion columns solely in fractions 6 and 7 (Fig. 5a,b) and had similar size profiles (Fig. 5c,d). qPCR analyses revealed that the OS EV fractions had more abundant *HSATI* and *HSATII* DNA (Fig. 5e), as observed with the PEG-isolated EVs of the same samples (Fig. 5f). Similarly, *HSATI* and *HSATII* levels were higher in the same OS versus control sera when normalized to EV concentration (Supplementary Fig. 9A and B). Furthermore, nucleic acids obtained from arbitrarily selected non-EV fractions from one control and one OS fractionation (Fig. 5g,h) revealed no detectable *HSATI* and only minimal *HSATII*, which was not higher in OS samples (Fig. 5i,j). Thus, *HSATI* and *HSATII* DNAs co-purified with EVs in SEC with a greater abundance in OS compared to control sera, similar to that of PEG preparations.

We next assessed whether OS-associated repetitive element DNAs co-purified with EVs in exosome CD9 or CD81 immunoaffinity capture. In pilot studies, we confirmed that our immunocapture approach enriched for exosomes by single particle interferometric reflectance imaging sensing (SP-IRIS) using an ExoView instrument^45^. SP-IRIS analyses showed that a similar number of EV particles eluted from CD9 immunoaffinity capture from control (21,006 particles, n = 1) and OS sera (21,264 ± 374 (SEM) particles, n = 2), that the eluted particles could be re-immunocaptured on the microarray-based solid phase chip coated with antibodies to exosomal surface markers (Fig. 5k,l), and that the re-captured particles were from 50 to 80 nm diameter (characteristic of exosomes) (Fig. 5m,n) and expressed various combinations of exosomal markers CD81, CD63, and CD9, similar to the PEG precipitated EV preparations of the same samples (Supplementary Fig. 10). Nanoparticle tracking analyses of the CD9 and CD81 immunoaffinity capture eluates showed particle size distributions similar to that of PEG precipitations but larger than reported by SP-IRIS (Fig. 5o,p, Supplementary Fig. 11A and B), as expected^46^. However, in contrast to PEG- and SEC-isolated EVs, CD9 and CD81 immunoaffinity captured EV preparations showed no significant difference in *HSATI* and *HSATII* DNA abundance between control and OS samples (Fig. 5q,r and Supplementary Fig. 11C). Thus, the OS-associated *HSATI* and *HSATII* DNAs failed to co-purify with EVs during immunocapture in contrast to their co-purification with EVs isolated via PEG precipitation or SEC. It is inferred that OS-associated *HSATI* and *HSATII* DNAs either fail to bind CD9 + or CD81 + exosomes or dissociate from such exosomes under immunocapture conditions.

### Enrichment of human satellite sequences in EV-associated DNA but not in total cfDNA in OS patient sera

**Figure 6:**
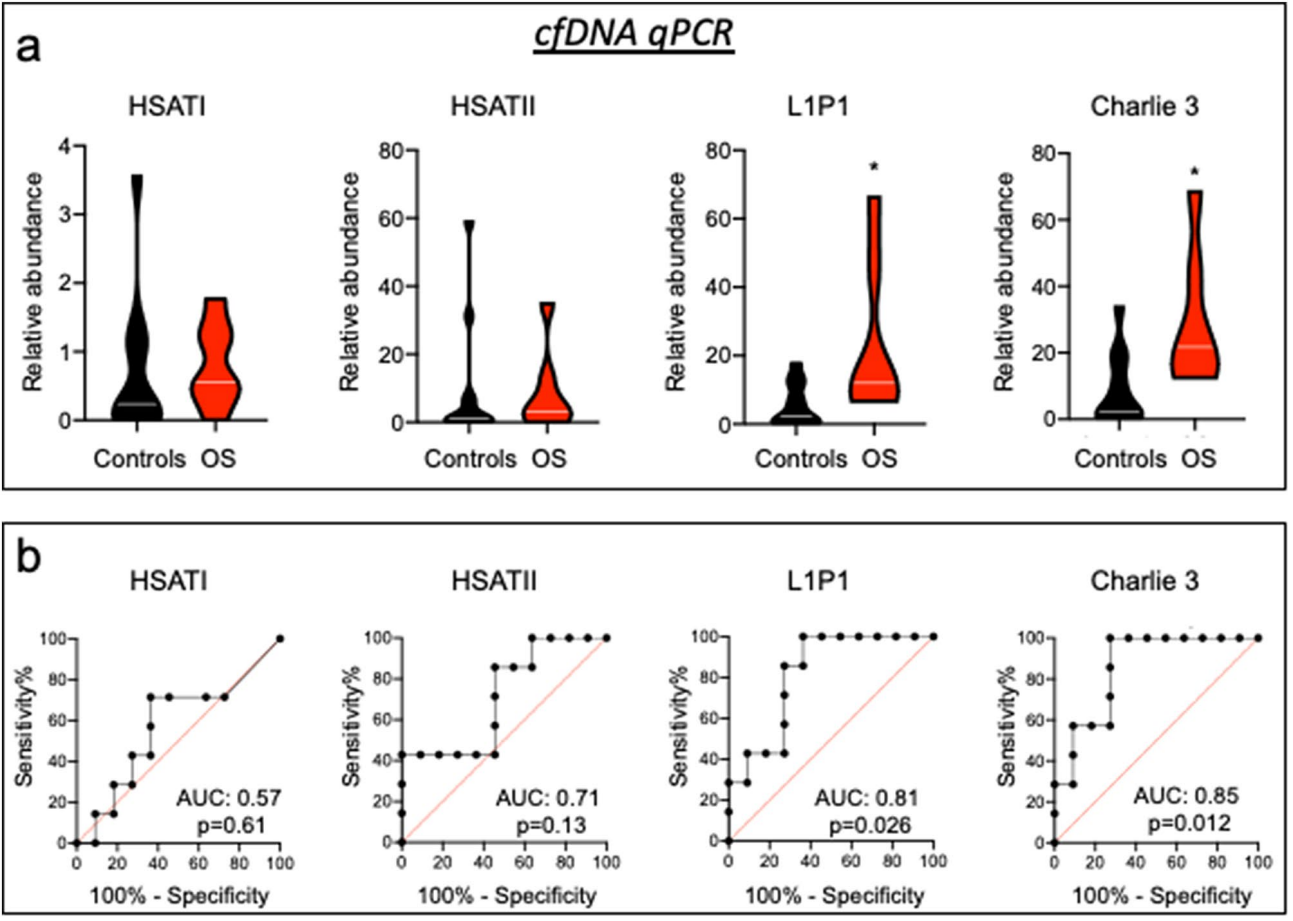
Human satellite sequences not enriched in total cfDNA in OS patient sera. (**a**) Violin plots representing relative abundance of *HSATI*, *HSATII*, *L1P1* and *Charlie 3* by qPCR of control (n = 11) and OS (n = 7) whole serum. White lines represent median. (**b**) Diagnostic value of *HSATI*, *HSATII*, *L1P1* and *Charlie 3* in OS patient whole serum. ROC curves were generated using data in (**a**). Groups were compared using two-tailed, unpaired, Mann Whitney U test; *p < 0.05.

Our finding that repetitive element DNAs were increased in OS patient PEG and SEC EV preparations yet not tightly bound to CD9+ or CD81+ exosomes raised the possibility that repetitive element DNAs might be more abundant in total cfDNA of OS patients and were proportionately present as contaminants in OS and control EV nucleic acid preparations. To examine this possibility, nucleic acids were extracted directly from equal volumes of OS and control sera using the same miRNeasy micro RNA extraction kit as used for EV preparations and the repetitive element abundance was examined by qPCR. This revealed that *L1P1* and *Charlie 3* were significantly more abundant whereas *HSATI* and *HSATII* were present at similar levels in OS and control cfDNA samples (Fig. 6a). Importantly, omitting the PEG EV preparation step eliminated the diagnostic sensitivity of *HSATI* and *HSATII* (AUC =  < 0.72) while reducing that of *L1P1* (AUC = 0.81) and not affecting that of *Charlie 3* (AUC = 0.85) (Fig. 6b) as compared to the AUC values obtained after PEG precipitation (Fig. 3c). These data imply that EV enrichment by PEG or SEC was required in order to detect the increased representation of *HSATI* and *HSATII* and to increase the sensitivity for *L1P1* in OS patient versus control sera.

## Discussion

Sensitive biomarkers are needed to detect incipient OS tumors and enable life-saving interventions in predisposed individuals. Prior studies identified a variety of potential OS biomarkers yet none had sufficient sensitivity to enable reliable OS detection^12,13^. To identify new OS biomarkers, we investigated nucleic acid sequences associated with circulating EVs in OS patients. This revealed an over-representation of diverse repetitive element sequences, among which human satellites *HSATI* and *HSATII* were the most significantly increased upon mapping to the GRCh38 and hg19 genome builds. The over-represented repetitive element sequences were confirmed in a validation cohort and found to reflect repetitive element DNAs that co-purified with circulating EVs but were not tightly bound to CD9 + or CD81 + exosomes. *HSATI* and *HSATII* were distinguished from other repetitive elements in that they were enriched in serum EV preparations but not in total cfDNA, implying that they segregated into distinct complexes in the circulation of OS patients.

Our detection of increased repetitive element DNAs in OS patient sera was enabled by a novel screening approach. First, in the discovery cohort, we prepared serum EVs using a precipitation method that concentrates exosomes as well as other EVs and non-vesicular constituents^29,30^, which enlarged the population of biomarker candidates. Second, we did not treat EVs or nucleic acid preparations with DNase, which enabled isolation of DNAs as well as RNAs and further diversified the potential biomarker pool. Third, we isolated nucleic acids using small RNA preparation kits that also captured repetitive element DNAs, and we built our sequencing library by direct ligation of adapters to extracted DNAs as well as RNAs, using an activity with properties similar to T4 RNA ligase^47^, which allowed the discovery of differentially represented DNA as well as RNA species. Finally, we evaluated repetitive element sequences that include diverse satellite and non-satellite categories that may comprise more than two-thirds of the human genome^48^. Repetitive elements are often ignored in human sequencing studies because of the complexity involved in properly aligning short sequencing reads to highly repetitive regions as well as poor understanding of their functional relevance^33^. However, the paucity of over-represented single copy sequences in OS patient EV preparations prompted us to consider whether repetitive element sequences might be over-represented.

To examine differential repetitive element sequence representation, we initially used RepeatMasker to align sequence reads against the Repbase library of known repeats^32^. This revealed an over-representation of all repetitive element categories in OS serum EV preparations, with the *LINE1* family member *L1P1* as the most significantly over-represented species (Supplementary Fig. 2). To more accurately identify differentially represented repetitive elements, we used TEtranscripts, which assigns both uniquely and ambiguously mapped reads to all possible gene and transposable element-derived transcripts in order to statistically infer the correct gene or transposable element abundances^34^. TEtranscripts analyses confirmed that repetitive elements were over-represented in OS serum EV preparations and identified *HSATI*, *HSATII* and *Charlie 3*, among others, as significantly over-represented (Table 2). Different elements were identified when reads were aligned to GRCh38 or to hg19, likely due to the presence of alternative repetitive-element-enriched sequence assemblies in GRCh38^35^.

We validated the increased abundance of selected repetitive element sequences in a second patient cohort. In the validation set, we observed their increased abundance via qPCR, without reverse-transcription and in a DNase-sensitive manner, implying differential representation of repetitive element DNAs. Repetitive element DNA sequences were similarly increased in OS samples from USA (CHLA) and China (HLOH) and did not correlate with the OS grade, suggesting that these elements are produced independently of OS type. EV-associated repetitive element DNAs showed a high sensitivity and specificity for sera of patients with an OS diagnosis, with a significant AUC > 0.9 for *HSATI*, *HSATII* and *L1P1*. However, the sensitivity was diminished by omitting the EV preparation step, particularly for *HSATI* and *HSATII* (Fig. 6), suggesting that these OS-associated repetitive element DNAs are segregated from bulk cfDNA.

At present the physical state of the repetitive element DNAs that enables their enrichment from OS patient sera is unknown. Prior studies detected repetitive element DNAs in growth medium of human OS cells, yet their physical state was not characterized^49^. In serum EV preparations, the OS-associated repetitive element DNAs were sensitive to DNase I, indicating that they were not a protected EV cargo (Fig. 4a), in contrast to DNAs in cultured cancer cell-derived microvesicles^27^. As repetitive element DNAs co-purified with EVs in PEG precipitation and SEC but not in CD9 or CD81 immunoaffinity capture, they might associate with exosomes too weakly to survive immunocapture, associate with CD9- and CD81-negative exosomes, associate with non-exosome EVs, or participate in large non-vesicular complexes that co-purify with EVs in the PEG and SEC methods. Despite prior reports that DNA is present in cancer exosomes or small EVs^27,50–52^, a recent reassessment demonstrated that small vesicles are not vehicles of active DNA release and that double stranded DNA was associated with non-vesicular entities that are extruded from cancer cells^53^. Similar to our results, this reassessment did not observe DNA associated with immunoaffinity capture of CD81-positive exosomes, further supporting that exosomes do not contain DNA or tightly associate with other particles that contain DNA. Understanding the biogenesis of repetitive element DNAs that are enriched in OS patient EV preparations could provide insight into OS pathogenesis as well as a tool with which to detect asymptomatic OS or other conditions in which EV-associated repetitive element DNAs are elevated.

A limitation of this study is that it is based on two relatively small patient cohorts and examined a mixture of EV-associated DNAs and RNAs in the discovery cohort. Thus, the sensitivity of the repetitive element DNA analyses for OS and the specificity for OS versus other cancers may be refined by defining differential DNA representation in a larger series of OS as well as other cancers and non-cancerous conditions. Nevertheless, the results suggest that EV-associated repetitive element DNAs are among the most sensitive markers of newly diagnosed OS identified to date, with ROC curve AUCs of 0.90 for *HSATI* and 0.97 for *HSATII* (Fig. 3c). By comparison, in prior OS biomarker analyses, circulating miRNAs had at best ROC curve AUCs of 0.833–0.955, yet were significantly elevated in only a subset of many similar miRNA screens^11,54–56^. Likewise, a deep sequencing approach detected circulating tumor DNA aneuploidy in only 50% of treatment-naïve OS patients^12^. Still, in this study, the increased repetitive element DNAs were observed in sera of already-diagnosed OS patients, and further refinements are likely to be required in order to detect repetitive element DNAs produced by smaller, incipient tumors prior to their clinical appearance.

A final question raised by our findings is whether similar EV-associated repetitive element DNAs are increased in the circulation of patients with other cancers. Notably, centromeric and pericentric repetitive element RNA sequences, particularly alpha satellites and satellite II and III sequences, were reported to be overexpressed in testicular, liver, ovarian, and lung cancers compared to corresponding normal tissues^57^. The pericentric human satellite II (*HSATII*) RNA was reported to be the most differentially expressed satellite subfamily in pancreatic cancer tissue and was also overexpressed in lung, kidney, ovarian, colon and prostate cancers^58,59^. Moreover, *HSATII* was one of the six most up-regulated satellite sequences in a study comparing fresh bone and OS samples by RNA-seq^28^. *LINE-1* was also overexpressed in pancreatic and prostate tumor samples^60,61^. However, although *LINE1* and other repetitive element RNAs were detected in cancer cell-derived EVs in culture^27^, their up-regulation has not been reported for circulating cell-free RNA in cancer patients. Our detection of the enriched DNA templates of these repetitive element RNAs in the OS patient circulation raises the possibility that circulating repetitive element DNAs may also be enriched in additional cancer types.

## Materials and methods

### Patients and samples

This study was reviewed and approved by the institutional review board at Children’s Hospital Los Angeles (approval no. CCI-13-00223) and at Henan Luoyang Orthopedic Hospital (approval no. 2015-01). All participants gave a written informed consent. Parents/Legally authorized persons gave informed consent on behalf of the all minors and subjects above 14 years old gave assent. All analyses were conducted in accordance with relevant guidelines and regulations. Blood samples were collected during a clinically indicated venipuncture from previously untreated patients with primary diagnosis of OS and from volunteer subjects with no known medical conditions, i.e. healthy controls. Control sera for the validation cohort were obtained from local volunteer subjects and from Innovative Research Inc. (Novi, MI, USA). Blood was drawn in serum separator collection tubes (SST), clotting was allowed for 30 min at room temperature in vertical position and then tubes were centrifuged at 1000*g* for 10 min at 4 °C. Serum was collected, immediately aliquoted, and stored at − 80 °C.

### Discovery cohort: EV isolation, nucleic acid extraction, and sequencing

Serum EVs were isolated using ExoQuick (System Biosciences Inc. (SBI), Mountain View California, USA) and aliquots frozen. One aliquot was used for NTA analyses and on confirmation of high EV purity aliquots were thawed and nucleic acid extracted using SeraMir (SBI) without DNase treatment, according to manufacturer instructions. The sequencing library was constructed using TailorMix miRNA Sample Preparation (SeqMatic) with a selection of small nucleic acids from 140 to 300 bases. 5′-RNA adapters and 3′-DNA adapters (SeqMatic, personal communication) were directly ligated to nucleic acid substrates, followed by PCR amplification. Libraries were sequenced to generate single-end 50 bp reads on MiSeq 500 platform (Illumina).

### Validation cohort: EV isolation and nucleic acid extraction

Serum was cleared by centrifugation at 3000 × *g* for 15 min at 4 °C. For polyethylene glycol (PEG) precipitation, 50–200 ul of cleared serum was combined with an equal volume of freshly prepared 16% PEG 6000 (Sigma-Aldrich) in 1 M NaCl, to give a final concentration of 8%, incubated for 30 min on ice, centrifuged in a tabletop microfuge at 16,000 × *g* for 2 min at room temperature (Eppendorf, model 5424 R using an FA-45-24-11 fixed angle rotor) and the pellet resuspended in a volume of PBS equal to that of the starting serum volume. For size exclusion chromatography (SEC), ~ 300 ul of cleared supernatant was centrifuged at 10,000* g* for 30 min at 4 °C in a fixed angle rotor and loaded onto a glass Econo-column (Bio-Rad, 10 cm height, 1.5 cm diameter) packed with Sephacryl S-300 High Resolution (GE Healthcare) and pre-washed with 0.32% Sodium Citrate in PBS. The cleared serum was allowed to enter the resin by gravity flow and eluate collected in 20 fractions of 15 drops (~ 500 ul) on a Model 2110 Fraction Collector (Bio-Rad). For each fraction, the protein concentration and the presence of EVs was characterized by Bradford method (Bio-Rad) and nanoparticle tracking analysis (see below), respectively. EV fractions were concentrated on a 100 kDa Amicon ultra centrifugal filter (Millipore) from 2 × 500 µl to a final volume of ~ 100 ul. Immunoaffinity capture of CD81 + or CD9 + EVs was carried out using the Exo-Flow Exosomes Purification Kit (SBI, Mountain View California). Briefly, 200ul of cleared serum was precipitated with 200ul of 16% PEG 6000 as above, and the pellet re-suspended in 200ul of PBS. 50 ul of this EV preparation were incubated in 20 μl of anti-CD81 or anti-CD9 pre-coated magnetic beads (9.1 μm) on a rotating rack at 4 °C overnight. CD81 + or CD9 + EVs were eluted from the beads in the Exosome Elution Buffer at 25 °C for 30 min.

Nucleic acids were extracted from 20 to 200 ul of EV preparations (or from 50 ul of serum) using miRNeasy Micro kit (Qiagen) and suspended in 14 ul of RNase/DNase-free H_2_O (depending the initial volumes of serum) according to the manufacturer’s instruction. For samples intended for reverse-transcription, a spike-in control (*C. elegans *miR-39-3p) miRNA mimic (Qiagen) was added (1.6 × 10^9^ copies) after the lysis step. Nucleic acid size and concentration were analyzed on an RNA Pico 6000 chip using an Agilent Bioanalyzer (Agilent, Palo Alto, CA, USA), equipped with Expert 2100 software, which generated an electrophoretic profile and the corresponding ‘pseudo’ gel of the sample. After separation, nucleic acid sizes were normalized to a 25 bp RNA marker. Samples showing nucleic acids of > 200 bp were eliminated from the study.

### Particle size and concentration measurement by nanoparticle tracking analysis

EV preparations were analyzed by nanoparticle tracking using a NanoSight NS300 (Malvern, Worcestershire, U.K.) configured with a high sensitivity sCMOS camera (OrcaFlash2.8, Hamamatsu C11440, NanoSight Ltd). In brief, each sample was mixed by vortexing, and subsequently diluted in particle-free PBS to obtain a concentration within the recommended measurement range (10^8^–10^9^ particles/mL), corresponding to dilutions from 1:100 to 1:500. After optimization, settings were kept constant between measurements. Ambient temperature was recorded manually and did not exceed 25 °C. Approximately 20–40 particles were in the field of view for each measurement. Three videos of 30 s duration were recorded for each sample. Experiment videos were analyzed using NTA 3.2 Dev Build 3.2.16 software (Malvern).

### Single particle interferometric reflectance imaging sensing (SP-IRIS)

EVs from PEG and immunocapture preparations were analyzed on ExoView R100 platform (Nanoview Biosciences, MA). Briefly, EVs within these preparations were immunocaptured on a multiplexed microarray chip with CD9, CD81 CD63, and CD41a antibody spots, as well as negative control IgG antibody spots to determine the level of non-specific binding, and then probed for CD9, CD81, CD63, and CD41a surface markers with respective additional fluorescent antibodies. EVs from PEG preparation and eluted EVs from immunoaffinity were diluted in solution A (Nanoview Biosciences, MA). The samples were incubated on the ExoView Tetraspanin Chip (EV-TC-TTS-01) placed in a sealed 24-well plate for 16 h at room temperature. The chips were then washed three times in 1 ml PBST for 3 min each on an orbital shaker. Then, chips were incubated with ExoView Tetraspanin Labeling ABs (EV-TC-*AB*-01) that consist of anti-CD81 Alexa-555, anti-CD63 Alexa-488, and anti-CD9 Alexa-647. The antibodies were diluted 1:5000 in PBST with 2% BSA. The chips were incubated with 250 µL of the labeling solution for 2 h. The chips were then washed once in PBST, three times in PBS followed by a rinse in filtered deionized water and dried. Immunocaptured EVs on the microarray chip were imaged on a single EV-basis with the ExoView R100 reader using the nScan2 2.9 acquisition software. The data were then analyzed using the NanoViewer 2.9 software (Nanoview Biosciences, MA) that counts and sizes fluorescent nanoparticles immunocaptured on the antibody spots. For exosome analysis the size window was selected to include particle sizes from 50 to 200 nm.

### Reverse transcription (RT) and qPCR

Equal volumes of nucleic acid prepared as above were reverse-transcribed using iScript cDNA Synthesis Kit (Bio**‐**Rad) in 20 ul volume according to the manufacturer's protocol. 0.5 ul of the samples produced with or without the RT step were analyzed in 10 ul qPCR reactions with iQ Green Supermix (Bio**‐**Rad) on an ABI 7900 Fast Real**‐**Time PCR System (Applied Biosystems) with the following cycling parameters: 94 °C, 30 s; 59 °C, 15 s; 68 °C, 25 s for 35 cycles. Relative sequence abundance was determined by the ΔΔCt method. In most PCR runs, a negative control with no nucleic acid template was added and never generated PCR product. PCR primers were designed manually to have a melting temperature of 58 °C and to generate amplicons of ~ 100 bp or as previously described for *HSATI*^62^ and *HSATII*^37^ (Supplementary Fig. 4) and obtained from Integrated DNA Technologies:

*HSATI* F: 5′-TAATGTGTGGGCTTGGGATT-3′, *HSATI* R: 5′-TGCATATGGAAAATACAGAGGCTA-3′ (amplicon: 406 bp); *HSATII* F: 5′- ATTCGATTCCATTCGATGATGATTCC-3′, *HSATII* R: 5′-GGAACCGAATGAATCCTCATTGAATG-3′ (prominent amplicons; 85, 134, 183 and 281 bp); *L1P1-orf2* F: 5′- ATCAGAGAATACTACAAACACCTCTAC-3′, *L1P1-orf2* R: 5′- AGAGTGTATGTGTCGAGGAAT-3′ (amplicon: 83 bp); *Charlie 3* F: 5′-ACAAAAGCACTGAAAAGCCTGC-3′, *Charlie 3* R: 5′-TCCAGTCTACTCCGTAATCTCGT-3′ (amplicon: 104 bp); *HECDT2* F: 5′-TGTGAAAGACTTTCAGGAAGATGTAGAAAAA-3′, *HECDT2* R: 5′-GAGGGAGTGGCATCTTTCTTAAATG -3′ (amplicon: 131 bp). For *ZNF3* and *IL17RA*, TaqMan primers were used (*IL17RA*: Hs01285262_cn and *ZNF3*: Hs03631848_cn (Applied Biosystems) and PCR reactions were performed according the manufacturer’s instructions.

### DNase I and RNase A treatment of EV preparations

Intact EV preparations were treated with DNase I (Qiagen) in RDD buffer for 15 min at room temperature and then inactivated for 10 min at 70 °C. Intact EV preparations were treated with RNase A (Thermo Scientific) at final concentration 0.4 ug/ul with or without NaCl at final concentration 1 M for 10 min at 37 °C^39^ and inactivated by RNase inhibitor (Takara) at final concentration 2u/ul.

### RNA-Seq data processing, alignment and analysis

Fastq files were aligned to GRch38 (for analysis 1) or hg19 (for analysis 2) using STAR using the parameters recommended for TEtranscripts (i.e. allowing for up to 100 alignments per read)^34^, and the resulting BAM files were processed using TEtranscripts to quantify both non-repetitive element and repetitive element abundance.

### Statistical analysis

Groups were compared using two**‐**tailed, unpaired, Mann Whitney U test (*p < 0.05, **p < 0.01, ***p < 0.001, ****p < 0.0001). All analyses were performed using Prism 8 software (GraphPad).

## Supplementary information

Supplementary Information.
