# Supplementary material for: Extracellular vesicle-associated repetitive element DNAs as candidate osteosarcoma biomarkers
Source: Sci Rep. 2021 Jan 8;11:94. doi: 10.1038/s41598-020-77398-z (PMC7794510; doi:10.1038/s41598-020-77398-z)
Supplement: Supplementary file 1 — Supplementary Information. [file 41598_2020_77398_MOESM1_ESM.pdf]

Supplementary information

Extracellular vesicle-associated repetitive element DNAs as candidate osteosarcoma biomarkers

Authors:

Linda Cambier, Kevin Stachelek, Martin Triska, Rima Jubran, Manyu Huang, Wuyin Li, Jianying Zhang, Jitian Li, David Cobrinik

Correspondence to:

Jitian Li, MD, PhD (Email: jitianlee@hotmail.com)

David Cobrinik, MD, PhD (Email: dcobrinik@chla.usc.edu)

Ref: Submission ID 67178d25-df82-4b63-a01e-b664b93a6db1

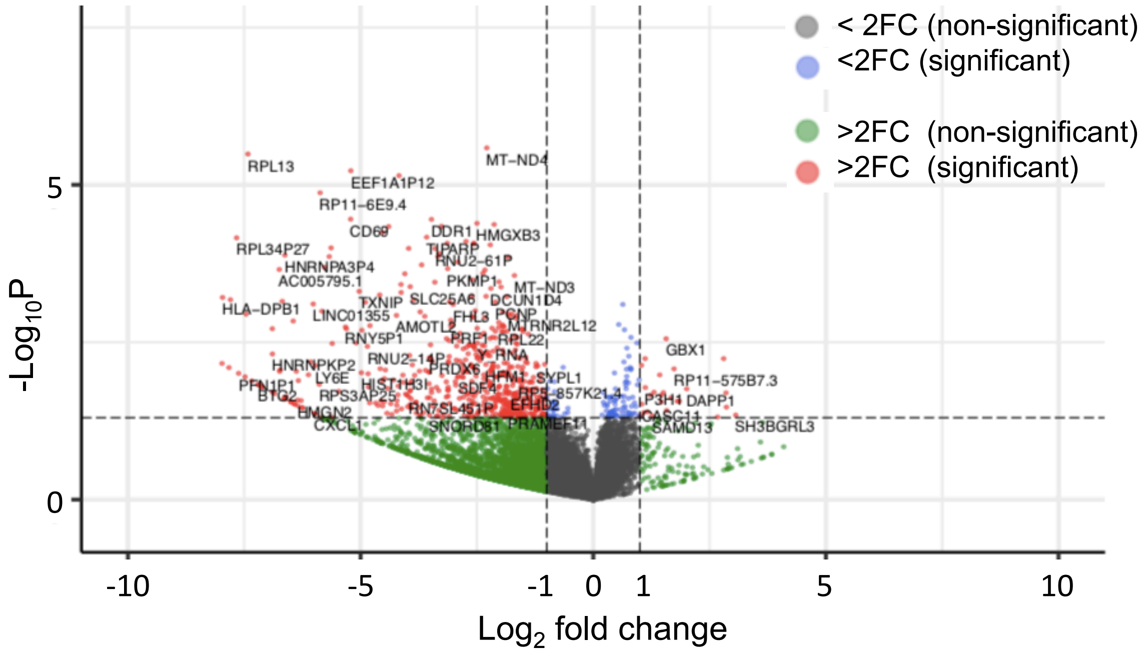

Supplementary Fig. 1: Differential representation of single copy nucleic acid sequences in OS versus control EV preparations. Volcano plot of the differentially represented features evaluated with DESeq2 between control and OS serum EV preparations. Single-copy genes represented significantly (FDR<0.05) and non-significantly (FDR>0.05) more than 2-fold in OS vs. control samples are shown in red and green, respectively. Those represented less than 2-fold in OS vs. control samples are shown in blue and grey.

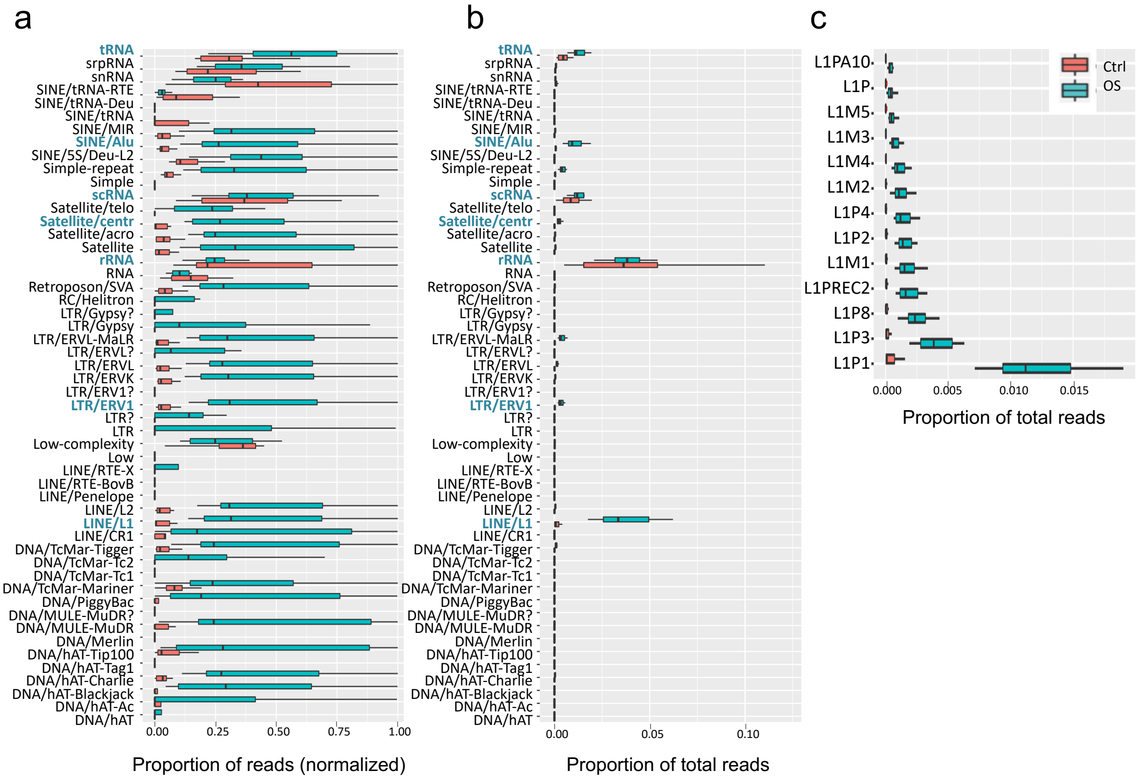

Supplemental Fig. 2: Differential representation of repetitive element sequences in OS versus control EV preparations. (a-c) Tukey box plots with mean (vertical line), 25th–75th percentiles (boxes), and maximum and minimum values up to a 1.5X the interquartile range (whiskers) for OS (green) and control (red) sequencing libraries aligned to RepeatMasker. (a) Proportion of reads aligned to different repetitive element classes in OS and control EV preparations normalized to the sample with maximum abundance in the class. (b) Proportion of reads aligned to each repetitive element category. (c) Proportion of reads aligned to each LINE1 subfamily.

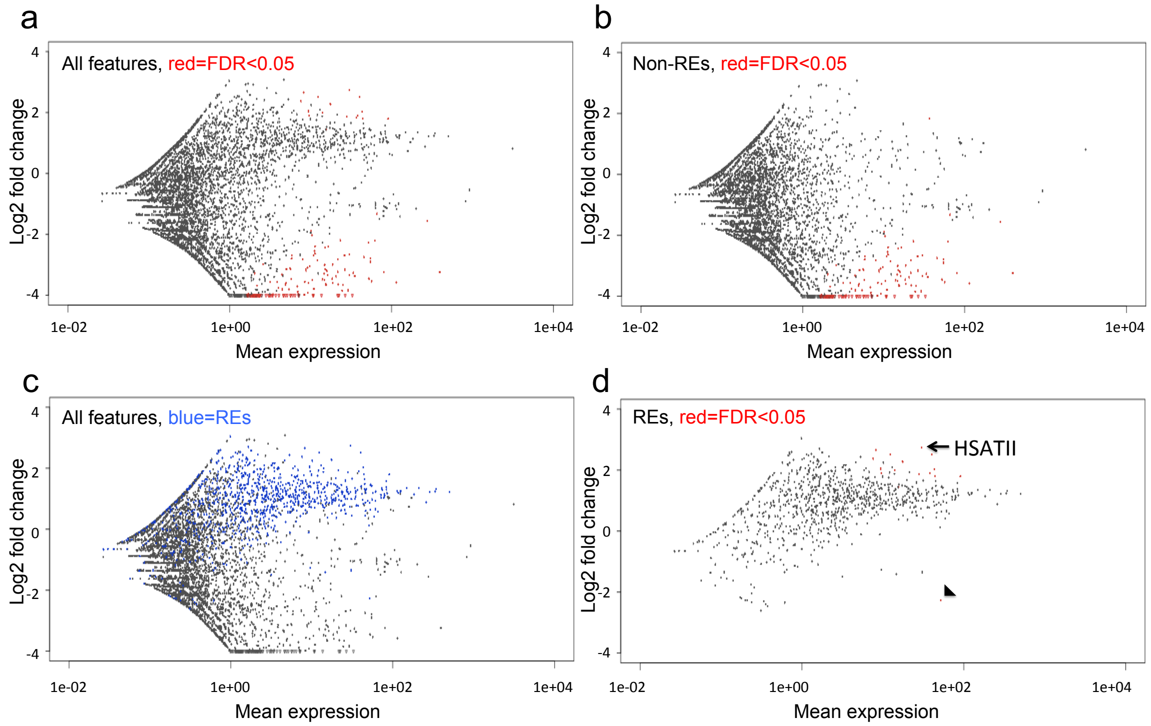

Supplementary Fig. 3: Over-representation of repetitive elements in OS EV–associated sequences. MA plot for the differentially represented sequences between control and OS serum EV preparations as defined by TEtranscripts using genome build hg19. (a) Differentially represented single-copy genes and repetitive elements (REs), significantly differentially represented in red. (b) Differentially represented single-copy genes, significantly differentially represented in red. (c) Differentially represented single-copy genes in black and REs in blue. (d) Differentially represented REs, significantly differentially represented in red. Arrow, the significantly over-represented *HSATII*. Arrowhead, significantly under-represented RE. Significantly differentially represented: FDR<0.05, Wald test.

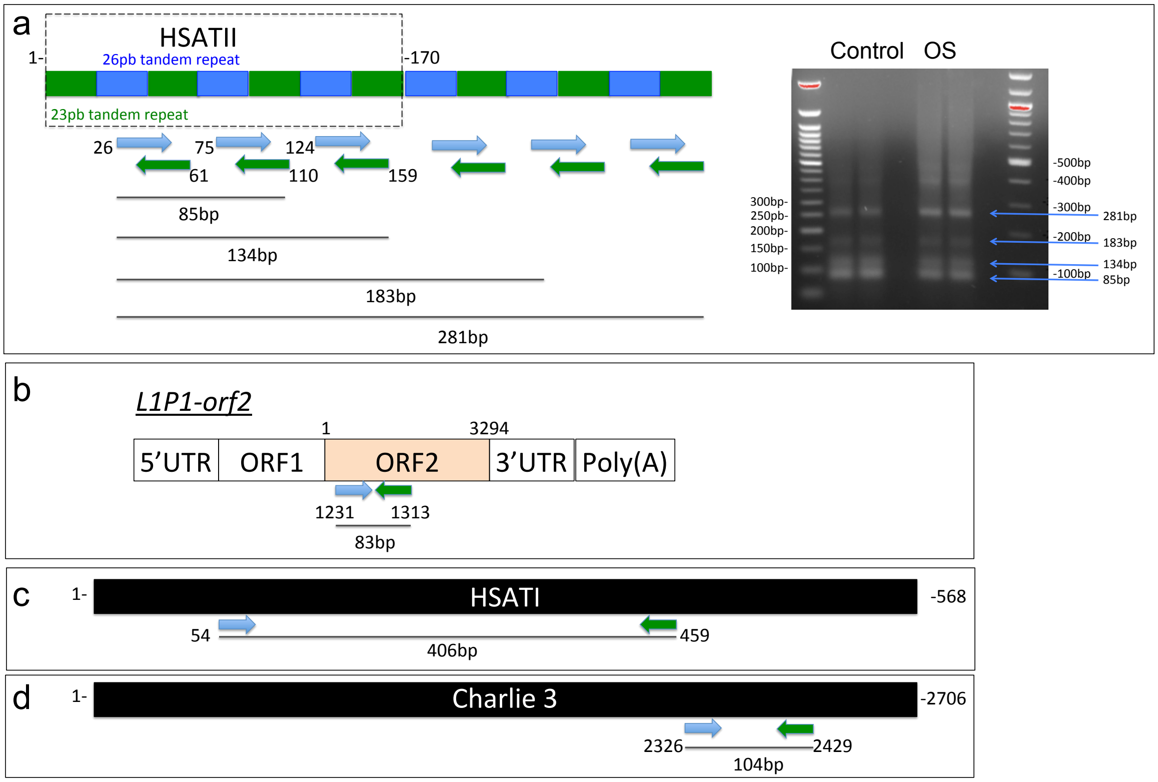

Supplementary Fig. 4: Structure of repetitive elements examined by PCR. (a, left) *HSATII* gene composed of tandem repeats of 23 and 26 nucleotides. Blue and green arrows represent forward and reverse primers; respectively. (a, right) Electrophoresis gel of *HSATII* amplification in control and OS samples in duplicate. Arrows, bands of indicated sizes that were removed for sequencing. (b) *L1P1* gene structure. Primers amplify a region of *ORF2*. Blue and green arrows represent forward and reverse primers; respectively, with their position indicated from the first nucleotide of *ORF2*. (c, d) Position of primers for *HSATI* and *Charlie 3*. Grey lines represent the amplicon for each repetitive element.

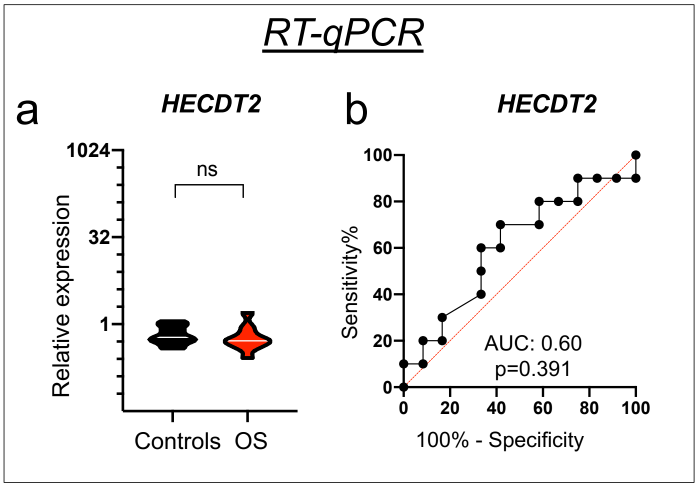

Supplementary Figure 5: No differential representation of single-copy genes in OS compared to control EV preparations. (a) Violin plots of relative representation of *HECDT2* by RT-qPCR in control (n=12) and OS (n=10) serum EV preparations. RT-qPCR was normalized against *C. elegans* external spike-in miR-39–3p RNA added during nucleic acid extraction. White lines represent median. (b) Diagnostic value of *HECDT2* in OS serum EV preparations. ROC curves were generated using data in (a). Groups were compared using two-tailed, unpaired, Mann Whitney U test; ns: P >0.05.

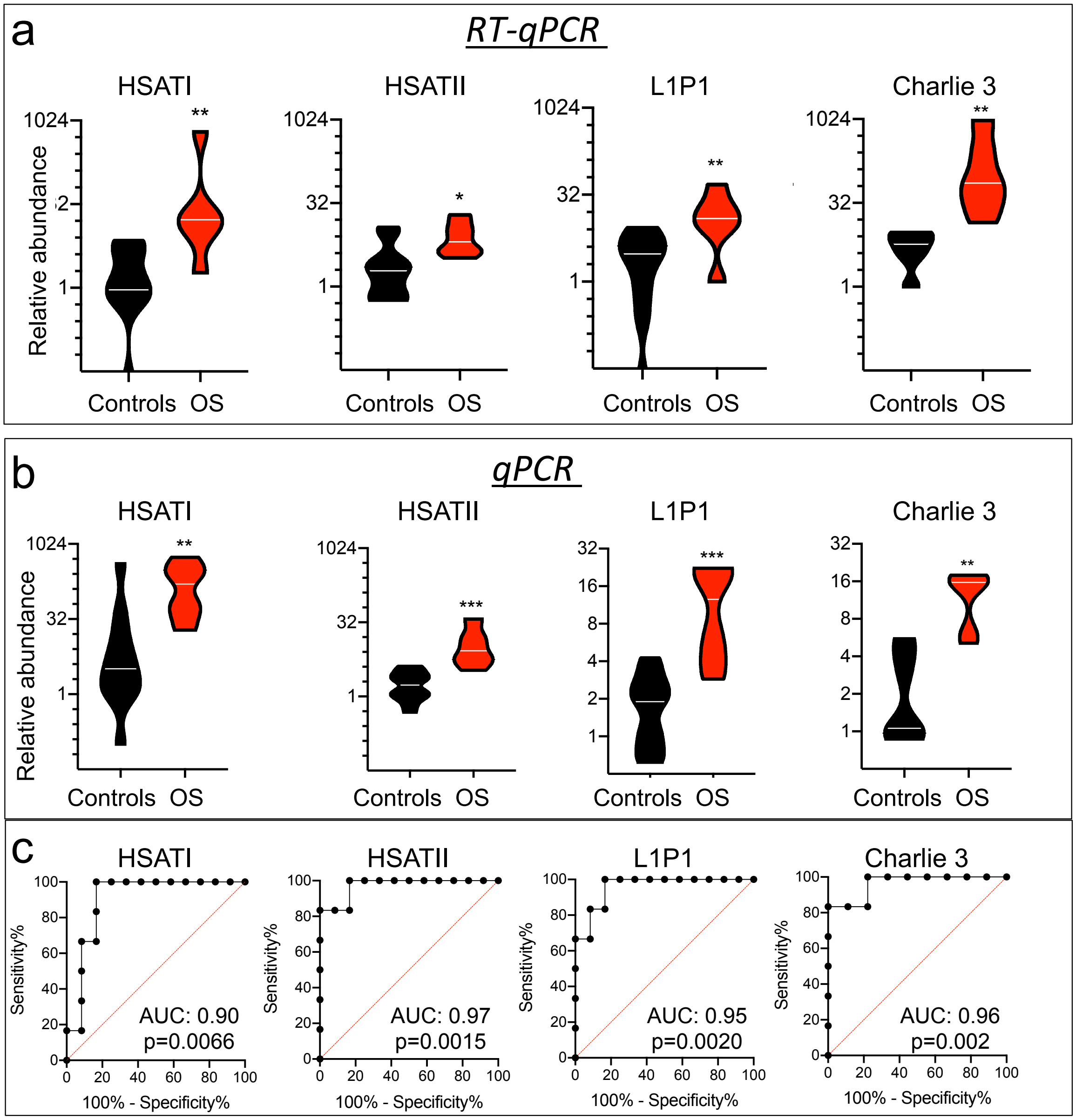

Supplementary Figure 6: Over-representation of repetitive elements in OS compared to control EV preparations in a validation cohort. Re-evaluation of the results for the validation cohort in Figure 3, omitting OS1 and OS3 samples also present in the discovery cohort.

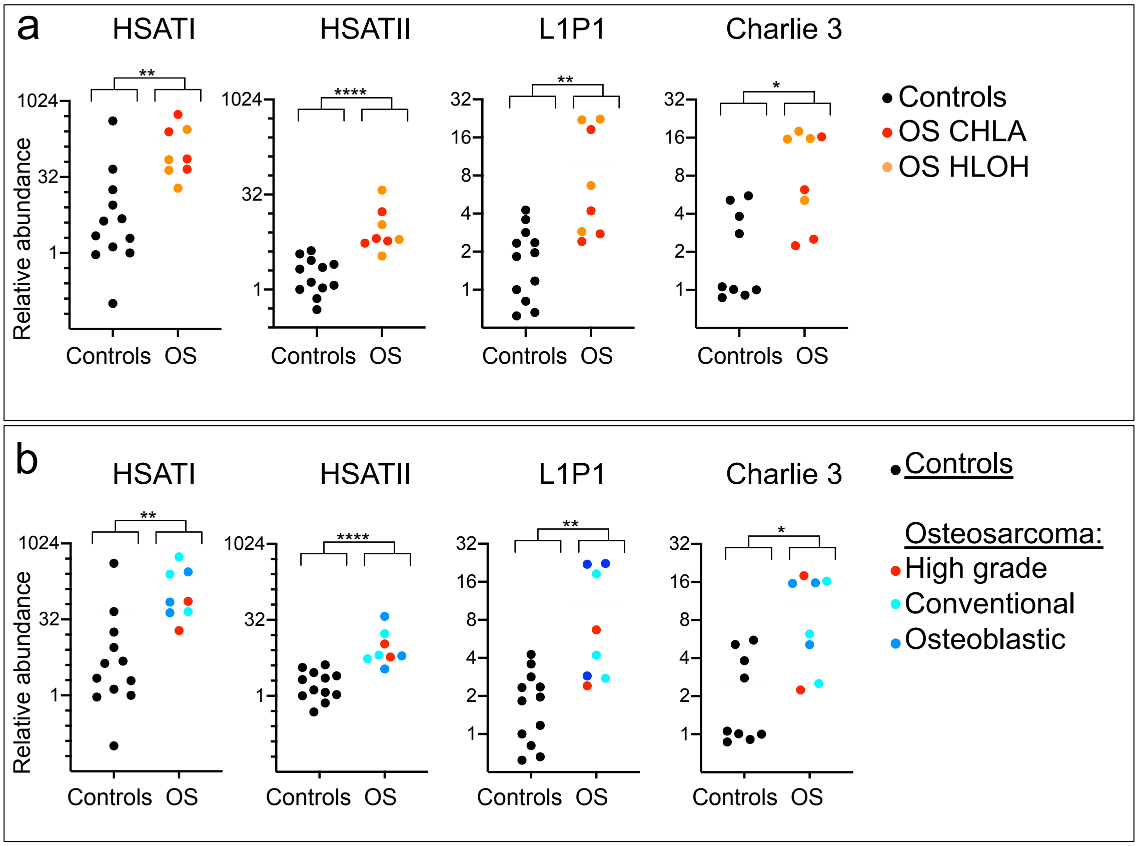

Supplementary Figure 7: Repeat element abundance relative to ethnicity and OS type. (a) Scatter plots representing relative abundance of *HSATI*, *HSATII*, *L1P1* and *Charlie 3* DNA similar to violin plots in Figure 3C with each individual sample represented by colored dots according the OS sample source (CHLA and HLOH). (b) Scatter plots representing relative abundance of *HSATI*, *HSATII*, *L1P1* and *Charlie 3* DNA similar to violin plots in Figure 3C with each individual sample represented by colored dots according the OS type. Groups were compared using two-tailed, unpaired, Mann Whitney U test; *P <0.05; **P <0.01; ***P < 0.001.

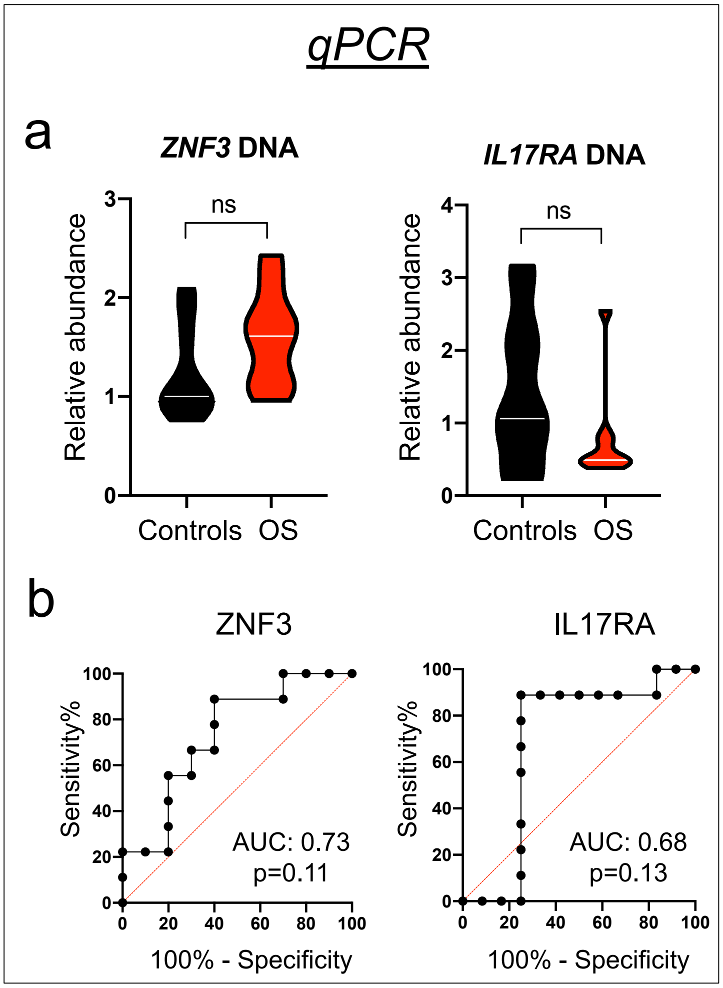

Supplementary Figure 8: No differential abundance of single-copy genes in OS compared to control EV preparations. (a) Violin plots representing the relative abundance of *ZNF3* and *IL17RA* DNA by TaqMan assay, in the absence of reverse transcription, in control (n=12) and OS (n=8) serum EV preparations. qPCR was performed on equal proportions of nucleic acid extracted from PEG precipitations of 200ul of OS and control sera. White lines represent median. (b) Diagnostic value of *ZNF3* and *IL17RA* in OS serum EV preparations. ROC curves were generated using data in (a). Groups were compared using two-tailed, unpaired, Mann Whitney U test; ns: P >0.05.

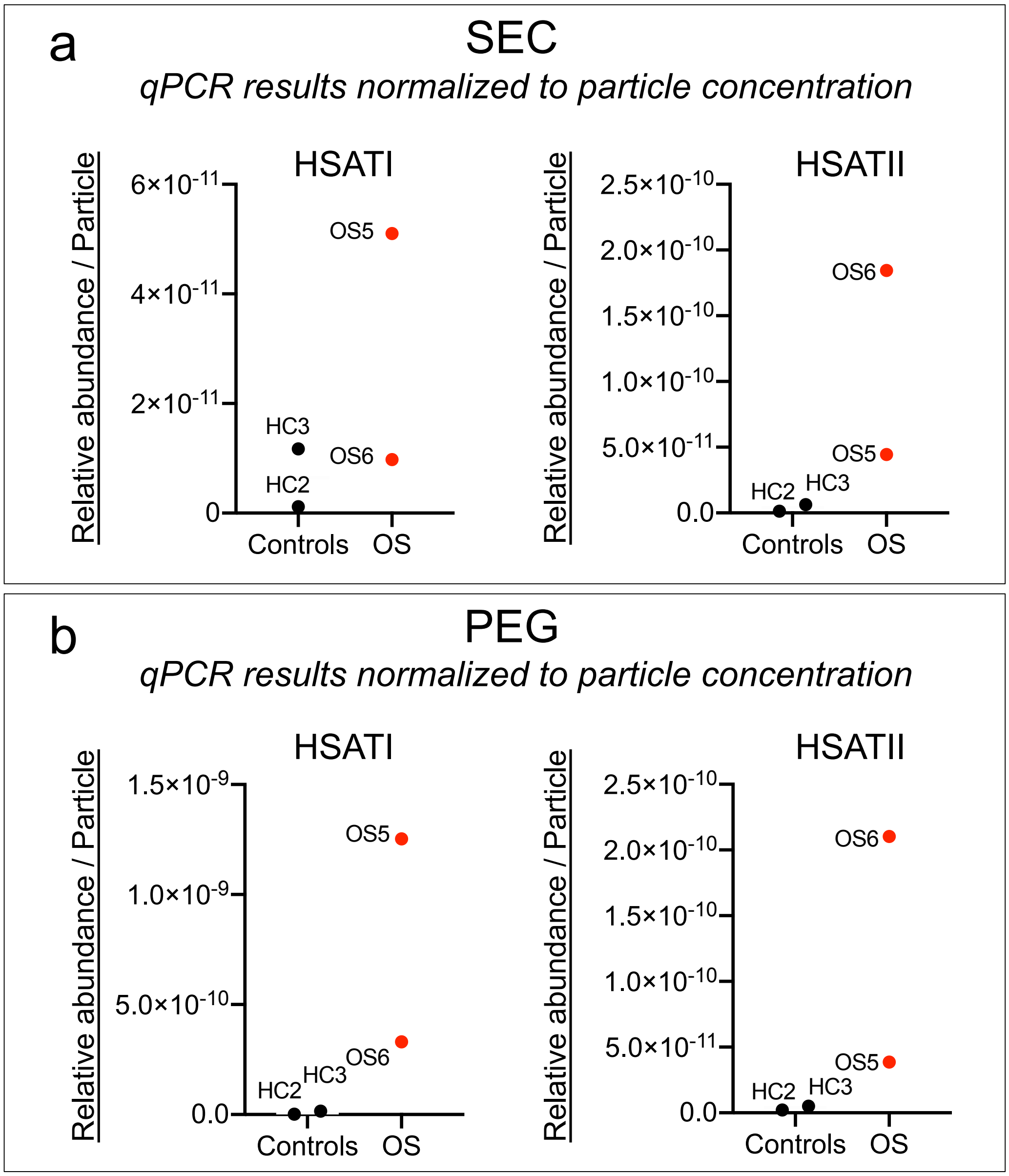

Supplementary Figure 9: Abundance of repetitive elements DNAs co-purified with PEG precipitation and size exclusion chromatography (SEC) and normalized to particle concentration. Relative abundance of *HSATI* and *HSATII* DNA in two control and two OS SEC (a) and PEG (b) EV preparations was defined using the same qPCR reactions as shown in Figure 5e-f (performed on equal proportions of nucleic acids extracted from PEG-precipitated or SEC-isolated EV preparations from 200ul of OS and control sera), but with abundance normalized to the particle concentration of each sample.

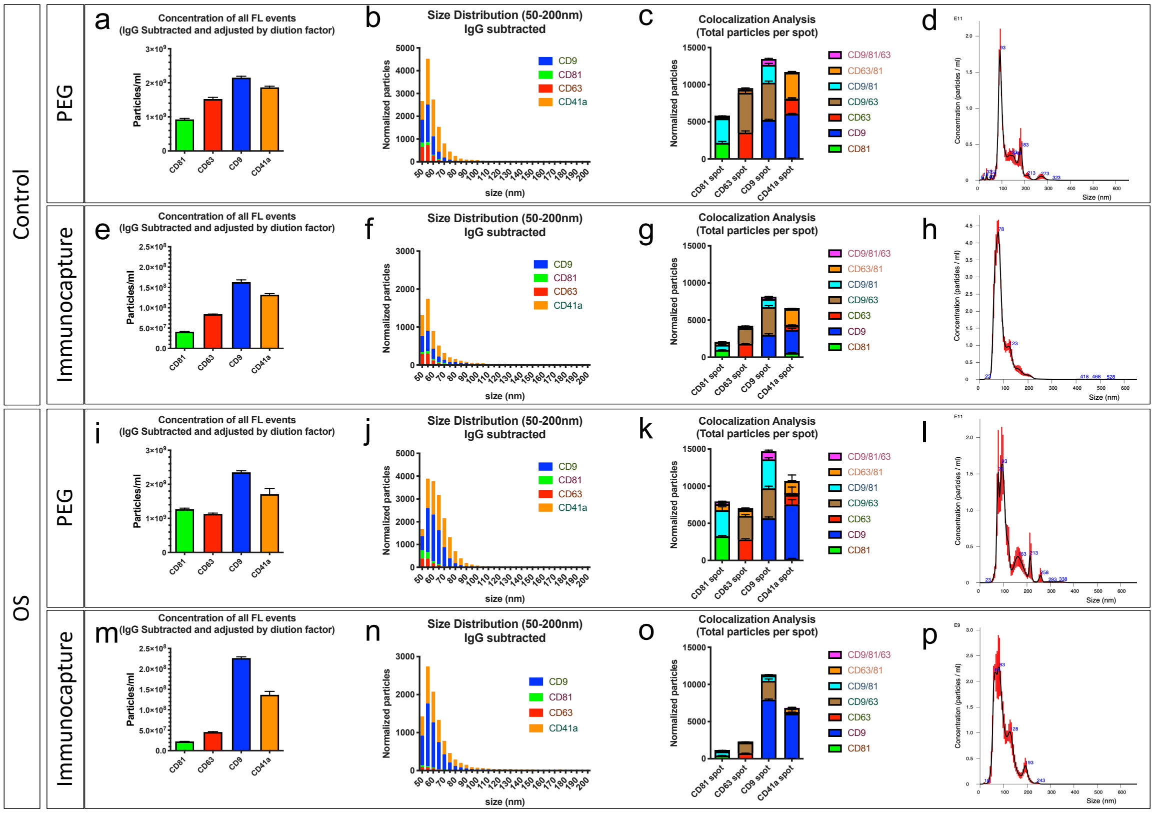

Supplementary Figure 10: Phenotyping of EVs isolated by PEG precipitation and CD9 immunoaffinity capture.

SP-IRIS analyses by ExoView on control (top rows) and OS (bottom rows) samples obtained by PEG precipitation (a-c and i-k) and by CD9 immunoaffinity capture (e-g and m-o). (a, e, i, m) Concentration of EV particles captured on the ExoView CD9, CD81, CD63, and CD41a antibody spots as measured by intrinsic fluorescence. Results depict the mean of the measurement of triplicate spots ± SEM, subtracted for IgG spot values and adjusted by dilution factor. (b, f, j, n) Representative size distribution of label-free EV particles immunocaptured on the CD9, CD81, CD63, and CD41a antibody spots. Results depict the mean of the measurement of triplicate spots ± SEM, subtracted for IgG spot values. (c, g, k, o) Immunophenotyping of EV particles on the CD9, CD81, CD63, and CD41a antibody spots determined using fluorescent antibodies. (d, h, l, p) Control and OS EVs isolated by PEG precipitation (d and l) and CD81 immunoaffinity capture (h and p) and analyzed by nanoparticle-tracking. Panels e, m, f, and n are identical to Fig. 5k, l, m, and n, respectively.

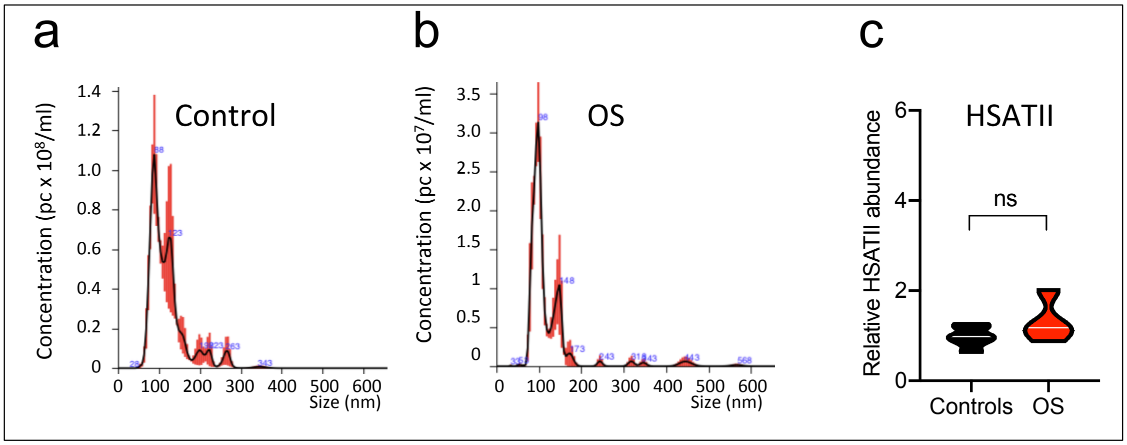

Supplementary Figure 11: No co-purification of OS-associated repetitive element DNAs with EVs by CD81 immunoaffinity capture. (a, b) Size distribution and particle number of control (a) and OS (b) EVs isolated by CD81 immunoaffinity capture and analyzed by nanoparticle-tracking. (c) Violin plot representing abundance of *HSATII* of control (n=6) and OS (n=4) immunoaffinity capture of CD81-positive exosomes evaluated by qPCR. White lines represent median. Groups were compared using two-tailed, unpaired, Mann Whitney U test; ns, not significant, p <0.05.
